# First-time exploitation of Pranlukast's intrinsic fluorescence: a novel cetrimide-enhanced spectrofluorimetric platform for pharmaceutical, plasma, and content uniformity analysis

**DOI:** 10.1039/d5ra05505a

**Published:** 2025-10-09

**Authors:** Omkulthom Al kamaly, Lateefa A. Al-Khateeb, Michael K. Halim, Galal Magdy, Ahmed Emad F. Abbas

**Affiliations:** a Department of Pharmaceutical Sciences, College of Pharmacy, Princess Nourah bint Abdulrahman University P.O. Box 84428 Riyadh 11671 Saudi Arabia; b Department of Chemistry, Faculty of Science, King Abdulaziz University P.O. Box 80203 Jeddah 21589 Saudi Arabia; c Analytical Chemistry Department, Faculty of Pharmacy, October 6 University 6 October City Giza 12585 Egypt dr.ahmedeemad@gmail.com ahmed.emad.pha@o6u.edu.eg +20-1014077871; d Pharmaceutical Analytical Chemistry Department, Faculty of Pharmacy, Kafrelsheikh University Kafrelsheikh 33511 Egypt; e Department of Pharmaceutical Analytical Chemistry, Faculty of Pharmacy, Mansoura National University Gamasa 7731168 Egypt

## Abstract

A novel, eco-friendly, and highly sensitive spectrofluorimetric method has been developed and validated for the quantification of Pranlukast (PNK) based on its intrinsic native fluorescence without the need for derivatization or fluorescent labeling. The method employs micellar enhancement with cetrimide to achieve a strong fluorescence signal at *λ*_ex_ = 286 nm and *λ*_em_ = 418 nm. Linearity was obtained in the range of 100–800 ng mL^−1^ (*r*^2^ = 0.9993), with limits of detection (LOD) and quantification (LOQ) of 9.87 and 29.91 ng mL^−1^, respectively. The method demonstrated excellent precision (RSD% < 2.0), high accuracy (mean recovery 99.2–101.4%), and selectivity in pharmaceutical formulations and spiked human plasma, with an application range of 150–600 ng mL^−1^ encompassing the clinically relevant *C*_max_ (467 ng mL^−1^). Mechanistic investigations (fluorescence profiling, Stern–Volmer analysis, and lifetime measurements) confirmed that fluorescence enhancement arises from micellar incorporation and protection against collisional quenching. Moreover, a comprehensive greenness profile was assessed using multiple tools: NEMI (fully green), GEMAM (7.487), VIGI (80), CFA (0.002 kg CO_2_ per sample), and the RGBfast index (85), yielding a high overall sustainability score of 89%. The method contributes significantly to 11 UN Sustainable Development Goals (SDGs), offering a greener and more cost-effective alternative to conventional chromatographic procedures that typically rely on hazardous solvents and time-consuming steps. This work introduces the first spectrofluorimetric approach for PNK, offering advantages of simplicity, low cost, high sensitivity, and environmental sustainability compared to previously reported UV and chromatographic methods. The method's limitation lies in its reliance on a micellar medium, which may require optimization for other surfactants or biological matrices.

## Abstract 2

A cetrimide-enhanced spectrofluorimetric method provides rapid, sensitive, and eco friendly determination of Pranlukast in pharmaceuticals and plasma, validated with multiple sustainability tools and aligned with UN-SDGs.

## Graphical abstract

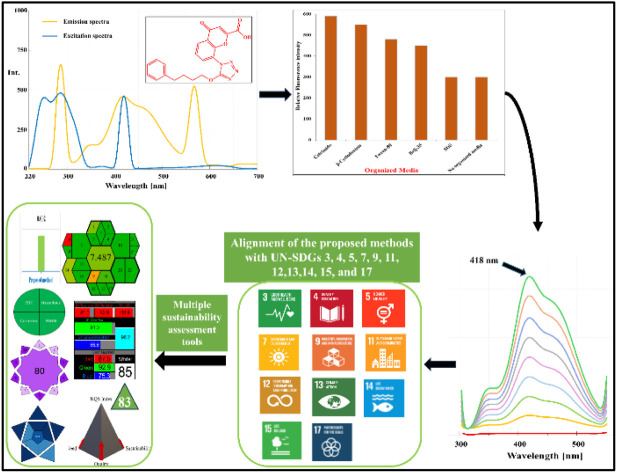

## 1 Introduction

Pranlukast (PNK) is a selective cysteinyl leukotriene receptor antagonist widely used in the management of bronchial asthma and allergic rhinitis, two of the most prevalent chronic respiratory and immunologic disorders, affecting hundreds of millions of individuals worldwide.^1^ Despite its clinical significance and increasing prescription in several regions, analytical methods for its determination remain scarce and underdeveloped. To date, only four analytical procedures have been reported for the quantification of PNK, each of which carries substantial limitations.

The first and most commonly cited method is based on high-performance liquid chromatography (HPLC), utilizing a mobile phase composed of acetonitrile and 0.1% glacial acetic acid (85 : 15 v/v).^2^ While this method provides reasonable separation, it relies on toxic organic solvents and lacks applicability to biological matrices such as plasma, thus rendering it unsuitable for pharmacokinetic or bioequivalence studies. Two additional methods based on liquid chromatography coupled with tandem mass spectrometry (LC-MS/MS) have been introduced for the determination of Pranlukast in human plasma to support pharmacokinetic profiling.^3,4^ These techniques employ on-line solid-phase extraction (SPE) systems and complex gradient elution protocols involving volatile buffers such as ammonium acetate and high percentages of acetonitrile. These techniques offer high sensitivity and selectivity but depend on sophisticated instrumentation, expensive reagents, and complex gradient elution protocols, rendering them impractical for routine use, particularly in developing or resource-limited settings. The fourth reported method, a UV-spectrophotometric technique,^5^ suffers from poor sensitivity and a narrow linear range (10–50 μg mL^−1^), making it incompatible with plasma studies where the expected *C*_max_ values of PNK are in the low nanogram per milliliter range (467 ng mL^−1^).^6^ These limitations underscore the critical need for a sensitive, selective, environmentally benign, and cost-effective analytical alternative.

In recent years, the advancement of green analytical chemistry (GAC) has garnered substantial attention, driven by the global shift toward environmentally responsible practices and the imperative to support the United Nations Sustainable Development Goals (UN SDGs).^7–9^ Analytical chemists are being called upon to minimize hazardous reagent usage, reduce waste generation, and replace energy-intensive techniques with more sustainable, resource-conscious alternatives. Within this context, spectrofluorimetry has emerged as a powerful tool in pharmaceutical and bioanalytical chemistry owing to its exceptional sensitivity, low detection limits, minimal reagent consumption, and rapid throughput.^10–12^ Numerous studies have successfully applied spectrofluorimetric methods for the determination of various drugs in bulk, dosage forms, and biological matrices, such as antihistamines, non-steroidal anti-inflammatory drugs, and cardiovascular agents.^13–15^ Furthermore, the use of native fluorescence, without derivatization or fluorescent tagging, offers an even greener approach by eliminating additional chemical modification steps that typically generate waste and involve toxic reagents.

**Fig. 1:**
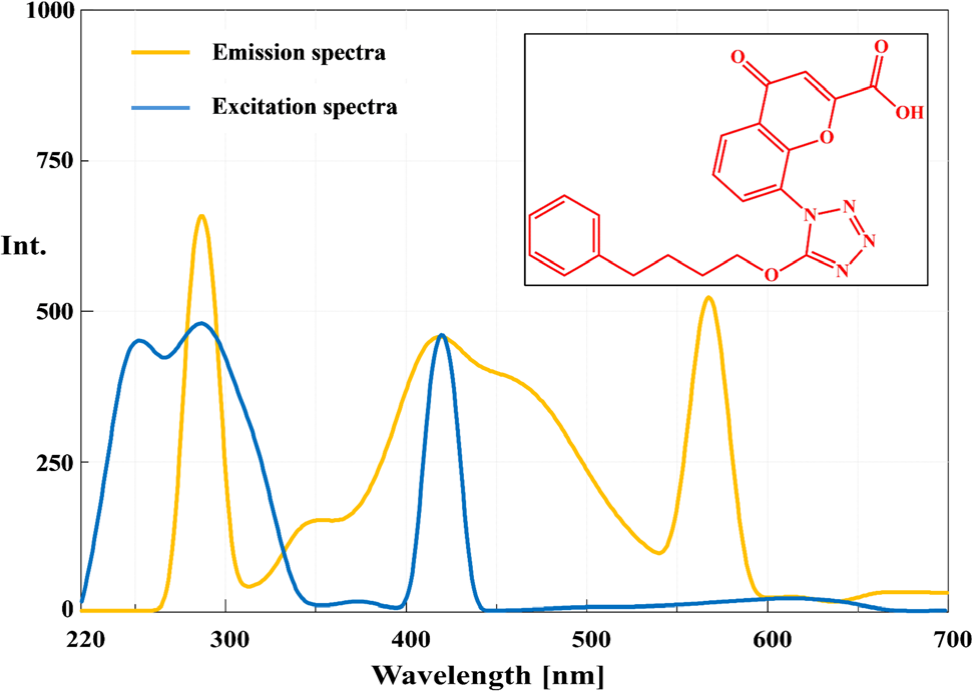
Chemical structure of PNK, excitation and emission spectra of PNK (500.0 ng mL^−1^) at optimum conditions.

Structurally, PNK is highly amenable to fluorescence-based quantification due to its conjugated aromatic system, including a substituted quinoline nucleus, aromatic ether linkages, and amide functionalities, all of which are known to support strong intrinsic fluorescence (Fig. 1). However, as with many hydrophobic drugs, its fluorescence in aqueous media may be limited by poor solubility and quenching effects from the surrounding environment. To overcome these limitations and further enhance fluorescence intensity, the current study employs micellar-enhanced spectrofluorimetry, a modern green strategy that harnesses supramolecular assemblies such as cationic surfactant micelles.^16–18^ Micelles can encapsulate hydrophobic drugs like PNK, shielding them from quenchers, improving solubilization, and altering the microenvironment around the fluorophore to increase quantum yield.^19,20^

To the best of our knowledge, no fluorimetric method has been reported for the determination of PNK. In this study, we report a native fluorescence-based spectrofluorimetric method for the quantification of PNK in pharmaceutical formulations and spiked human plasma. The method combines micelle-enhanced fluorescence with simple instrumentation to achieve nanogram-level sensitivity while maintaining eco-friendly and cost-effective operation.

The main advantages of the proposed method include its simplicity, affordability, high sensitivity, environmental sustainability, and applicability to complex biological matrices. Its innovation lies in being the first spectrofluorimetric approach applied to PNK, supported by comprehensive mechanistic investigations that explain the observed fluorescence enhancement. The principal limitation of the method is its reliance on micellar media, which may require optimization for other surfactants or biological environments.

Furthermore, in alignment with current sustainability evaluation trends, a comprehensive greenness and sustainability assessment was conducted using various validated tools, including the National Environmental Methods Index (NEMI), Greenness Evaluation Metric for Analytical Methods (GEMAM), Blue Applicability Grade Index (BAGI), Violet Innovation Grade Index (VIGI), carbon footprint analysis (CFA), and the Fast Red- Green-Blue (RGBfast) index, confirming its potential as a model for sustainable pharmaceutical analysis.

## 2 Experimental

### 2.1 Instrumentation and software

All fluorescence measurements were performed using a Cary Eclipse fluorescence spectrophotometer (Agilent Technologies, Santa Clara, CA, USA), equipped with a pulsed xenon flash lamp as the excitation source. The instrument parameters were optimized as follows: excitation and emission slit widths were set at 5 nm; detector voltage at 800 V; scan rate at 600 nm min^−1^; data interval at 1 nm; and a smoothing factor of 20 was applied to enhance the signal-to-noise ratio.

The optimal excitation and emission wavelengths for PNK were identified as 286 nm and 418 nm, respectively, based on systematic 3D excitation–emission matrix studies. Emission spectra were acquired in the range of 350–500 nm with automatic background subtraction and spectral correction for instrument response. All analyses were conducted at ambient temperature (25 ± 2 °C) using 1.0 cm quartz cuvettes (Hellma Analytics, Müllheim, Germany) containing a 3.0 mL sample volume.

Time-resolved fluorescence lifetime measurements were conducted using a time-correlated single photon counting (TCSPC) system with a NanoLED-286 pulsed excitation source (Horiba Scientific, Edison, NJ, USA) and a photomultiplier tube detector (Hamamatsu R928P).

Surface tension measurements for critical micelle concentration (CMC) determination were performed using a Du Noüy ring tensiometer (Krüss K6, Hamburg, Germany) with automatic temperature control. Temperature-dependent fluorescence studies were conducted using a thermostated cuvette holder with Peltier temperature control (Quantum Northwest, Liberty Lake, WA, USA) maintaining temperature stability within ±0.1 °C.

pH values were measured using a Jenway 3510 digital pH meter (Jenway, Staffordshire, UK) equipped with a combined glass electrode, calibrated daily using NIST-traceable standard buffers (pH 4.01, 7.00, and 10.01). Sample filtration was carried out using 0.45 μm hydrophilic PTFE syringe filters (Phenomenex, Torrance, CA, USA). All weighing was performed using an analytical balance (Sartorius CP225D, Göttingen, Germany) with a readability of 0.1 mg.

For quantum yield measurements, UV-visible absorbance was measured using a Cary 60 UV-vis spectrophotometer (Agilent Technologies, USA). Viscosity measurements for mechanistic studies were performed using an Ostwald viscometer (Schott Instruments, Mainz, Germany) calibrated with distilled water at 25 °C. All data acquisition and spectral processing were performed using Cary Eclipse software version 1.1 (Agilent Technologies).

Time-resolved fluorescence data analysis was conducted using DAS6 decay analysis software (Horiba Scientific), and mechanistic modeling was performed using Origin Pro 2023 (OriginLab Corporation, Northampton, MA, USA).

### 2.2 Materials and chemicals

#### 2.2.1 Reference standards

Pharmaceutical-grade PNK reference standard (purity 99.67%, lot number: 28925-1) was purchased from Cayman Chemical Company (Ann Arbor, MI, USA). The certificate of analysis was reviewed, and the standard was stored in a desiccator at 4 °C, protected from light and humidity, until use.

#### 2.2.2 Pharmaceutical formulations

Onon® capsules (label claim: 112.5 mg Pranlukast per capsule, batch numbers: ON240515, ON240617, and ON240711) manufactured by Ono Pharmaceutical Co., Ltd (Osaka, Japan), were obtained from a local authorized pharmacy. Capsules were stored as per the manufacturer's instructions (below 25 °C, protected from light and moisture) and analyzed within their shelf life. According to the manufacturer's declaration, the formulation contains Pranlukast as the active ingredient and the following inactive excipients: lactose monohydrate, microcrystalline cellulose, hydroxypropyl cellulose, and magnesium stearate. Three independent batches were tested to confirm reproducibility of the results.

#### 2.2.3 Solvents and reagents

Cetyltrimethylammonium bromide (cetrimide, ≥99% purity, lot number: MKBX4475V) was purchased from Sigma-Aldrich (St. Louis, MO, USA). Alternative surfactants including sodium dodecyl sulfate (SDS, ≥99%), polyoxyethylene (20) sorbitan monooleate (Tween-80, ≥99%), polyoxyethylene (23) lauryl ether (Brij-35, ≥99%), and β-cyclodextrin (≥97%) were obtained from Sigma-Aldrich for comparative studies. Solvents used included ethanol, methanol, acetonitrile, and ethyl acetate (all HPLC grade, ≥99.8%), obtained from Sigma-Aldrich, Seelze, Germany. Potassium iodide (KI, ACS reagent grade, purity ≥99.0%) for Stern–Volmer quenching studies was obtained from Fisher Scientific (Hampton, NH, USA). Glycerol (reagent grade, purity ≥99.5%) for viscosity studies was purchased from J. T. Baker (Phillipsburg, NJ, USA).

Britton–Robinson buffer (BRB) components including boric acid (H_3_BO_3_, ≥99.5%), orthophosphoric acid (H_3_PO_4_, 85%), and acetic acid (CH_3_COOH, ≥99.7%) were of analytical grade and obtained from Sigma-Aldrich. Sodium hydroxide (NaOH, ≥97%) was procured from Merck KGaA (Darmstadt, Germany). Quinine sulfate dihydrate (≥99%, lot number: Q1250) was obtained from Sigma-Aldrich and used as quantum yield reference standard. Ultrapure water (18.2 MΩ cm resistivity) was generated using a Milli-Q water purification system (Millipore, Bedford, MA, USA).

#### 2.2.4 Biological samples

Drug-free human plasma was obtained from VACSERA (The Egyptian Holding Company for Biological Products and Vaccines, Giza, Egypt). Samples were collected from healthy, fasting adult volunteers under protocols approved by the Institutional Ethics Committee of VACSERA (protocol number: VACSERA-EC/2024/017, approved in 2024) in compliance with the declaration of Helsinki. Plasma samples were collected in EDTA tubes, centrifuged at 4000 rpm for 10 min, and stored at −80 °C until analysis. All samples were processed within 4 hours of acquisition to maintain integrity. Only clear, non-hemolyzed plasma was used for spiked recovery studies to evaluate the method's applicability in bioanalysis.

### 2.3 Standard solution preparation

#### 2.3.1 Stock standard solution

A primary stock solution of PNK (1.0 mg mL^−1^) was prepared by accurately weighing 10.0 mg of the reference standard into a 10.0 mL volumetric flask, dissolving it in ethanol, and sonicating for 15 minutes at room temperature to ensure complete dissolution. The solution was stored in amber glass vials at 4 °C, protected from light. The stability of the stock solution was confirmed for 4 weeks under these storage conditions.

#### 2.3.2 Working standard solutions

Working solutions in the range of 100–800 ng mL^−1^ were freshly prepared on the day of analysis by appropriate dilution of the stock solution. In a 10.0 mL volumetric flask, an aliquot of the stock solution was transferred and mixed with 1.0 mL of freshly prepared 2.0% w/v aqueous cetrimide solution to create a micellar environment for fluorescence enhancement. The volume was then completed with water, whereby the ethanol content originating from the stock solution dilution did not exceed 2% v/v in the final solution. This trace amount of ethanol ensured adequate solubility of PNK while avoiding micellar destabilization or fluorescence quenching.

#### 2.3.3 Quantum yield reference solution

A reference solution of quinine sulfate (100 μg mL^−1^) was prepared by dissolving 10.0 mg of quinine sulfate dihydrate in 100.0 mL of 0.5 M sulfuric acid. Quinine sulfate in 0.5 M H_2_SO_4_ has a well-established fluorescence quantum yield of 0.546 ± 0.010 at 25 °C and was therefore employed as the reference fluorophore for determining the relative quantum yield of PNK. The solution was stored in amber bottles at 4 °C, protected from light, and used within one week of preparation to ensure photostability.

#### 2.3.4 Buffer solutions

BRB universal buffer (0.02 M) was prepared by mixing equal molar concentrations (0.04 M each) of boric acid, acetic acid, and orthophosphoric acid in double-distilled water. The desired pH values (2.0–12.0) were adjusted using 0.2 M sodium hydroxide, and the pH was confirmed with a calibrated pH meter. The buffer was fine-tuned as necessary and stored in polypropylene bottles at 4 °C. Buffers were prepared weekly to maintain freshness and avoid microbial contamination or pH drift.

#### 2.3.5 Surfactant solutions

A 10% w/v cetrimide stock solution was prepared by dissolving 10.0 g in 100.0 mL of double-distilled water under mild heating (40–50 °C) with constant stirring. Working solutions of varying concentrations (0.5%, 1.0%, 1.5%, 2.0%, 2.5%, and 3.0% w/v) were prepared by dilution with water. These solutions, along with other surfactants (SDS, Brij-35, Tween-80), were systematically investigated to assess their influence on PNK fluorescence and to identify the optimal micellar medium. All solutions were stored in airtight containers at room temperature (20–25 °C) and were stable for one month. The chosen concentration range was well above the reported critical micelle concentration (CMC) of cetrimide (∼0.033% w/v at 25 °C), thereby ensuring consistent micelle formation and reproducible fluorescence enhancement.

### 2.4 Determination of quantum yield

The relative quantum yield of PNK was determined using quinine sulfate in 0.5 M H_2_SO_4_ as the reference fluorophore, with a known quantum yield (*Φ*_ref_ = 0.546) of 0.546 at 25 °C. Equal volumes (0.1 mL) of PNK solution (100.0 μg mL^−1^ in methanol) and quinine sulfate solution (100.0 μg mL^−1^ in 0.5 M H_2_SO_4_) were each transferred into separate 10.0 mL volumetric flasks. The PNK flask was made up to volume with methanol. The quinine sulfate flask was made up to volume with 0.5 M sulfuric acid. UV-vis absorption spectra were recorded in the range 200–400 nm using 1.0 cm quartz cuvettes. Absorbance at the excitation wavelengths (312 nm for PNK and 350 nm for quinine sulfate) was measured in triplicate, ensuring that values remained below 0.1 absorbance units to minimize inner filter effects. Fluorescence emission spectra were recorded at the respective excitation wavelengths (312 nm for PNK; 350 nm for quinine sulfate), with emission collected from 350 to 600 nm. All measurements were conducted at 25 ± 1 °C.

The relative quantum yield was calculated using the following equation:^21^*Φ*_PNK_ = *Φ*_ref_ × (*I*_PNK_/*I*_ref_) × (*A*_ref_/*A*_PNK_) × (*η*_PNK_/*η*_ref_)^2^where: *Φ*_PNK_ is the quantum yield of PNK, *Φ*_ref_ is the quantum yield of quinine sulfate (0.546), *I*_PNK_ and *I*_ref_ are the integrated fluorescence intensities, *A*_PNK_ and *A*_ref_ are the absorbances at excitation wavelengths, and *η*_PNK_ and *η*_ref_ are the refractive indices of methanol (1.329) and 0.5 M H_2_SO_4_ (1.338), respectively.

### 2.5 Mechanistic studies

To elucidate the fundamental mechanisms governing the fluorescence enhancement of Pranlukast (PNK) in the presence of cetrimide, a comprehensive series of mechanistic investigations were conducted. The studies encompassed fluorescence intensity profiling as a function of cetrimide concentration, critical micelle concentration (CMC) determination, Stern–Volmer quenching analysis, time-resolved fluorescence measurements, UV-vis spectroscopic analysis, temperature and viscosity dependence studies, Job's plot analysis for stoichiometric evaluation, and partition constant assessment.^22,23^

#### 2.5.1 Fluorescence enhancement profile

To elucidate the micellar effect on PNK fluorescence, relative fluorescence intensity (RFI) was measured at a fixed concentration of PNK (500 ng mL^−1^) in the presence of cetrimide concentrations ranging from 0.00–3.00% w/v. Fluorescence emission spectra were recorded at *λ*_ex_ = 286 nm and *λ*_em_ = 418 nm. Each experiment was performed in triplicate, and mean values ± SD are reported.^24^

#### 2.5.2 Determination of critical micelle concentration (CMC)

The CMC of cetrimide under experimental conditions (aqueous-ethanolic medium with <2% v/v ethanol) was determined using dual approaches: (i) fluorescence intensity monitoring of PNK (200 ng mL^−1^) as a function of cetrimide concentration (0.00–0.10% w/v), and (ii) surface tension measurements using a Du Noüy ring tensiometer. The CMC was estimated from the inflection point of both plots, providing mutual validation of the critical concentration.^25^

#### 2.5.3 Stern–Volmer quenching studies

Quenching experiments were performed to investigate the protective effect of micelle incorporation against collisional deactivation.^26^ KI was employed as a collisional quencher. PNK solutions (300 ng mL^−1^) were prepared in (*A*) water and (*B*) 2.0% w/v cetrimide. Increasing concentrations of KI (0–200 mM) were added, and emission intensities were recorded. Stern–Volmer plots (*F*_0_/*F vs.* [*Q*]) were constructed, and quenching constants (*K*_sv_) were determined from the linear slopes according to the Stern–Volmer equation:^27^*F*_0_/*F* = 1 + *K*_sv_[*Q*]

#### 2.5.4 Time-resolved fluorescence (lifetime measurements)

Fluorescence lifetimes were measured by TCSPC using a NanoLED excitation source (286 nm) with emission monitoring at 418 nm.^28^ Samples of PNK (500 ng mL^−1^) were prepared in water and in 2.0% w/v cetrimide. Decay profiles were analyzed using monoexponential fitting algorithms, and average lifetimes (*τ̄*) were reported with *χ*^2^ ≤ 1.2 as the goodness-of-fit criterion.^29^

#### 2.5.5 UV-vis absorption spectra

UV-vis absorption spectra of PNK (10 μg mL^−1^) were recorded in the wavelength range 200–400 nm. Spectra were collected in (i) water and (ii) 2.0% w/v cetrimide to evaluate ground-state interactions and environmental polarity effects. Shifts in *λ*_max_ and changes in molar absorptivity were systematically compared.^30^

#### 2.5.6 Temperature and viscosity effects

Temperature-dependent fluorescence measurements were recorded at 10, 20, 30, and 40 °C in both aqueous and 2.0% w/v cetrimide media to assess thermal stability of the fluorescent species.^31^ For viscosity dependence studies, glycerol–water mixtures (0–40% v/v glycerol) were employed to investigate the role of molecular motion restriction. Emission intensities were normalized to the reference values at 20 °C and 0% glycerol, respectively.^32^

#### 2.5.7 Job's plot and partition constant determination

Continuous-variation (Job's) analysis was performed by systematically varying the mole fractions of PNK and cetrimide while maintaining a fixed total concentration of 1.0 mM. Fluorescence intensities were plotted against PNK mole fraction to assess stoichiometric complexation.^33^ The apparent partition constant (*K*_p_) was estimated from fluorescence intensity *versus* cetrimide concentration data using the partition equilibrium model:^34^*I* = *I*_0_ + (*I*_m_ − *I*_0_)(*K*_p_[*M*])/(1 + *K*_p_[*M*])where *I* represents the observed fluorescence intensity, *I*_0_ is the intensity in pure water, *I*_m_ is the intensity at micellar saturation, and [*M*] is the effective micelle concentration.

### 2.6 Calibration curve construction

To a series of 10.0 mL volumetric flasks, appropriate aliquots of the PNK stock solution were transferred to achieve final concentrations of 100–800 ng mL^−1^. To each flask, 1.0 mL of cetrimide solution (2.0% w/v in water) was added. The solutions were then diluted to volume with ethanol, ensuring the final aqueous content was below the threshold for micelle disruption. All solutions were thoroughly mixed and allowed to equilibrate at an ambient temperature (25 ± 2 °C) for 10 minutes to ensure full micellar formation and stabilization. Fluorescence measurements were conducted using excitation and emission wavelengths of 286 nm and 418 nm, respectively, with both excitation and emission slits set to 5 nm. A scan rate of 600 nm min^−1^ and 1 nm data interval were used, and a smoothing factor of 20 was applied. Triplicate readings were recorded for each concentration, and the mean RFI was plotted *versus* PNK concentration. Linear regression analysis was performed to derive the calibration equation, slope, intercept, and correlation coefficient (*r*^2^). Key analytical figures of merit, including sensitivity, limit of detection (LOD), and limit of quantitation (LOQ), were calculated according to ICH guidelines using standard equations.

### 2.7 Sample preparation and analysis

#### 2.7.1 Pharmaceutical dosage form analysis

Twenty Onon® capsules were individually weighed, and their average weight was recorded. Contents were emptied and homogenized. An accurately weighed amount equivalent to 10.0 mg PNK was transferred to a 100.0 mL volumetric flask and extracted with ∼50.0 mL ethanol by sonication (30 min). The solution was diluted to volume with ethanol, filtered through a 0.45 μm PTFE membrane, and appropriately diluted to prepare working solutions within the linear range (*e.g.*, 300–700 ng mL^−1^). Fluorescence analysis was conducted as described in Section 2.5.

#### 2.7.2 Content uniformity testing

The uniformity of dosage units was evaluated according to the British Pharmacopoeia (BP) guidelines.^35^ Ten individual capsules were analyzed using the same methodology described in Section 2.6.1. The content of each capsule was determined from the regression equation, and the acceptance value (AV) was calculated using the formula:AV = |*M* − *X̄*| + *ks*where *M* is the reference value (100% of label claim), *X̄* is the mean of individual contents, *k* is the acceptability constant (2.4 for *n* = 10), and *s* is the sample standard deviation. The batch passes the content uniformity test if AV ≤ 15.0.

#### 2.7.3 Plasma sample preparation and analysis

Drug-free plasma was thawed and gently vortexed. 0.5 mL plasma aliquots were spiked with PNK standard to yield concentrations equivalent to 4000–24,000 ng mL^−1^ prior to dilution. Protein precipitation was achieved with 3.5 mL cold ethanol (used instead of methanol to maintain solvent consistency with standard solutions), followed by vortexing (30 s) and centrifugation (10 000 rpm, 15 min, 4 °C). The supernatant was filtered through a 0.45 μm syringe filter. Aliquots of 1.0 mL of the filtrate were transferred to 10.0 mL volumetric flasks, followed by 1.0 mL of 2.0% w/v cetrimide. The volume was then made up with water (diluting residual ethanol from the stock) and allowed to equilibrate for 10 minutes. After a 40-fold dilution, final plasma concentrations ranged from 100 to 600 ng mL^−1^. Matrix-matched calibration curves were constructed, recovery was assessed by comparing the slopes of plasma and aqueous calibration plots, and matrix effects were quantified by comparing the fluorescence intensity of spiked plasma with pure standards. Plasma preparation followed previously published protocol^36^ with minor modifications.

### 2.8 Method validation

The proposed spectrofluorimetric method was validated in accordance with ICH Q2(R1) and ICH M10 bioanalytical guidelines to confirm its reliability for both pharmaceutical and biological applications. The validation process included evaluation of specificity, linearity, range, accuracy, precision, sensitivity, robustness, stability, and matrix effect.^37^

#### 2.8.1 Linearity and range

Calibration curves were constructed over the concentration range of 100–800 ng mL^−1^ using six levels in triplicate. Regression parameters, correlation coefficients, and residual analyses were determined.

#### 2.8.2 Sensitivity

The limits of detection (LOD) and quantification (LOQ) were calculated from the standard deviation of the intercept and the slope of the calibration curve, ensuring nanogram-level detectability.

#### 2.8.3 Accuracy and precision

Accuracy was assessed by spiking blank plasma samples and determining recoveries across multiple concentration levels. Precision was evaluated at three concentration levels (low, medium, high) using six replicates for intra-day and inter-day assays. Results were expressed as % RSD.

#### 2.8.4 Selectivity and matrix effect

Selectivity was examined by analyzing blank plasma and capsule excipients to confirm absence of interference. The matrix effect was assessed by comparing the slopes of plasma calibration curves with those from aqueous standards, with slope ratios used as indicators.

#### 2.8.5 Robustness and stability

Robustness was tested by small variations in experimental parameters (excitation wavelength ±2 nm, cetrimide concentration ±0.1%, ethanol proportion ±0.2%). Stability of PNK was investigated under short-term, long-term, and freeze–thaw conditions in plasma.

### 2.9 Statistical analysis

All data were expressed as mean ± standard deviation (SD). One-way analysis of variance (ANOVA) and Student's *t*-test were applied where appropriate. Regression analysis and residual plots were used to assess linearity and model fitting. Confidence intervals at the 95% level were calculated for calibration parameters. A *p*-value < 0.05 was considered statistically significant.

## 3 Results and discussion

PNK exhibits promising native fluorescence properties owing to its polyaromatic, conjugated, and amphiphilic structure. The molecule comprises multiple conjugated systems, including a substituted naphthalene ring, phenyl groups, and heteroaromatic moieties, along with polar functional groups such as carboxylic acid, amide, and ether linkages. These structural features contribute significantly to its intrinsic photophysical behavior, particularly in relation to π–π* and n–π* electronic transitions, intra-molecular charge transfer (ICT), and solvent–environment interactions.

### 3.1 Spectral characteristics and excitation–emission behavior

Spectral scanning of PNK revealed several excitation maxima at 224, 260, 286, and 349 nm. Among these, excitation at 286 nm produced the most intense and well-resolved emission band at 418 nm, with a large Stokes shift (∼132 nm) (Fig. 1). This shift is indicative of a stable excited state likely driven by ICT processes, wherein electron density is redistributed across the extended π-system upon excitation. The presence of electron-donating groups (*e.g.*, alkoxy) and electron-withdrawing groups (*e.g.*, carbonyl, carboxyl) on aromatic rings promotes ICT, resulting in long-wavelength emission. Such behavior is typical for aromatic fluorophores in rigid, planar conformations, which restrict vibrational relaxation and non-radiative decay.

This emission was highly reproducible in ethanol-based solutions, confirming that PNK possesses strong native fluorescence, making it a viable candidate for sensitive spectrofluorimetric determination.

### 3.2 Experimental parameters optimization

#### 3.2.1 Effect of diluting solvent

**Fig. 2:**
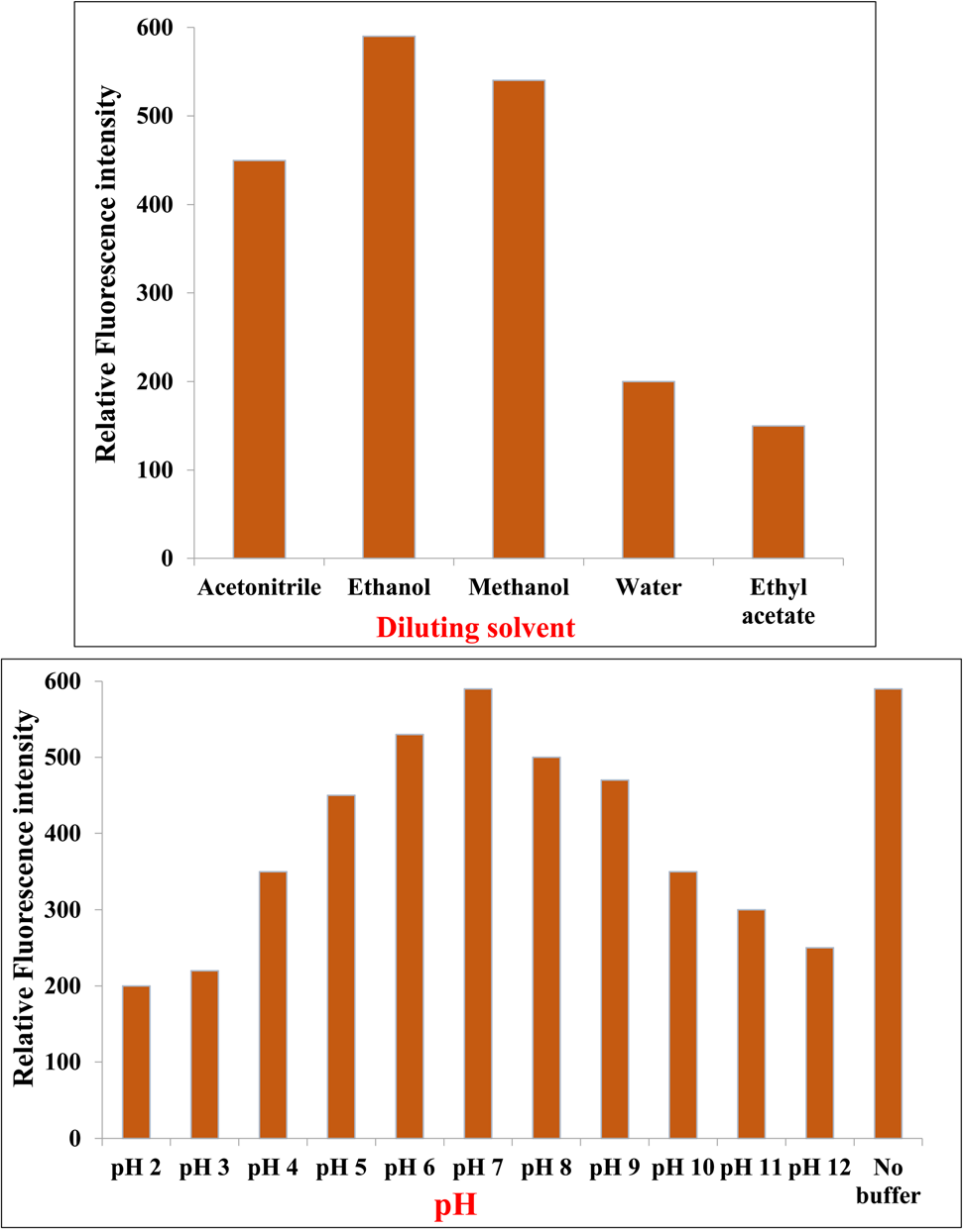
Effect of diluting solvent and pH on the relative fluorescence intensity of PNK (600.0 ng mL^−1^).

Solvent selection is critical in spectrofluorimetric methods, as solvent polarity, hydrogen bonding capacity, and dielectric constant can markedly influence fluorophore behavior. PNK was evaluated in various solvents including water, methanol, ethanol, acetonitrile, and ethyl acetate (Fig. 2).

Ethanol provided the highest fluorescence intensity. Its moderate polarity, protic nature, and good solubilizing capacity for both hydrophobic aromatic rings and polar functional groups allow for stabilization of both ground and excited states. Moreover, ethanol's ability to engage in mild hydrogen bonding likely minimizes non-radiative deactivation without significantly perturbing the excited state energy levels. This finding is consistent with previous reports indicating that ethanol enhances the emission of aromatic and heteroaromatic fluorophores by providing an optimal balance between polarity and hydrogen.^38–40^

Conversely: water, while green and highly polar, resulted in substantial fluorescence quenching, likely due to hydrogen bonding interactions with carbonyl and amide groups, which favor non-radiative relaxation. Similar quenching effects of water have been reported for structurally related drugs due to proton–donor interactions that destabilize the excited state.^41,42^ Methanol exhibited lower fluorescence than ethanol, possibly due to stronger hydrogen bonding and higher polarity, which may destabilize the excited state. Acetonitrile, being aprotic and polar, showed moderate fluorescence but lacked the ability to stabilize polar excited states *via* hydrogen bonding. Ethyl acetate produced minimal signal, likely due to poor solubility of the polar segments of PNK and low UV transparency. Thus, ethanol was selected as the optimal diluent for balancing solubility, fluorescence enhancement, and spectral clarity.

#### 3.2.2 pH effect

PNK contains several ionizable groups, including a carboxylic acid and amide moieties, which may affect its ionization state and, consequently, its fluorescence. The influence of pH was studied over a broad range (pH 2–12) using Britton–Robinson buffer (Fig. 2).

At low pH (2–4), the carboxyl group remains protonated, but potential protonation of the nitrogen atoms in heterocyclic rings or the amide group may disrupt conjugation or ICT, leading to decreased fluorescence. This trend agrees with earlier findings that protonation of heteroatoms often reduces conjugation and suppresses fluorescence.^43^

Between pH 5–7, the drug remains mostly in its neutral or zwitterionic state, maintaining maximum conjugation and optimal ICT, leading to the highest observed fluorescence. Comparable pH-dependent enhancement has been documented for other drugs with similar amphiphilic aromatic structures.^44,45^

Above pH 8, deprotonation of acidic moieties introduces negative charges, which can disturb the electronic distribution and promote solvent-excited state interactions, reducing fluorescence.

In strongly alkaline conditions, hydroxide ions can cause fluorescence quenching *via* collisional interactions or conformational instability.

Interestingly, ethanol-based solutions with no added buffer yielded comparable or even higher fluorescence intensity than pH 7 buffered solutions, confirming that protonation–deprotonation equilibria are minimized in non-aqueous media, and avoiding buffer avoids unnecessary ionic strength interference.

Hence, the method proceeded without buffer, using ethanol alone as a stable, interference-free medium.

#### 3.2.3 Effect of organized media

Organized media can significantly modulate fluorophore behavior by forming micellar or inclusion complexes that enhance fluorescence through microencapsulation, conformational rigidity, and shielding from quenchers.

**Fig. 3:**
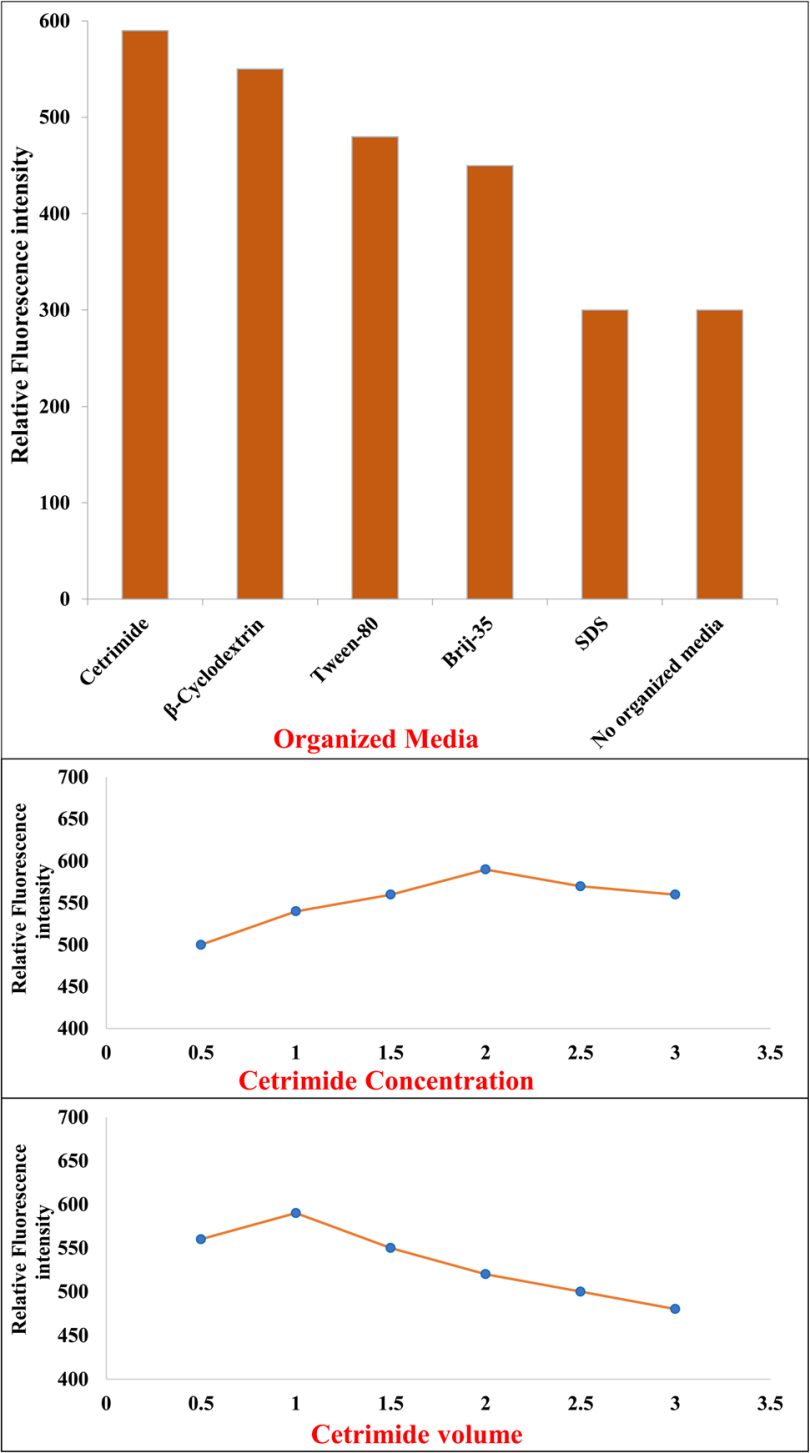
Effect of organized media, organized media volume, and organized media concentration on the relative fluorescence intensity of PNK (600.0 ng mL^−1^).

Five organized media were tested:

Cetrimide (cationic), SDS (anionic), Tween-80 and Brij-35 (nonionic), and β-cyclodextrin (neutral host molecule) (Fig. 3).

Cetrimide yielded the most significant enhancement (∼3-fold increase) in fluorescence intensity. As a cationic surfactant, cetrimide forms micelles that electrostatically attract the anionic or partially negative regions of PNK, increasing its residence in the micelle core. This hydrophobic microenvironment protects PNK's excited state from quenching by polar solvents and enhances rigidity, minimizing vibrational relaxation. This observation is consistent with previous studies where cationic micelles markedly improved fluorescence quantum yield by isolating fluorophores within a hydrophobic microdomain.^46,47^ SDS, being anionic, likely repels the negatively charged moieties of PNK, resulting in poor interaction and limited encapsulation, which explains the weak fluorescence response.^48^

Tween-80 and Brij-35, as nonionic surfactants, offer hydrophobic domains but lack specific electrostatic interactions with PNK. As a result, only moderate enhancement was observed, likely due to weaker drug incorporation and limited structural stabilization.

β-Cyclodextrin, a cyclic oligosaccharide, improved fluorescence moderately *via* host–guest complexation. PNK's hydrophobic aromatic moieties likely fit into the hydrophobic cavity of β-CD, shielding them from the aqueous phase. However, the inclusion is less efficient and dynamic compared to micelle formation by cetrimide. Comparable β-CD inclusion effects have been documented in enhancing fluorescence of hydrophobic drugs, though usually to a lesser extent than micellar encapsulation.^49,50^

Thus, cetrimide was clearly superior, and further studies were conducted to optimize its concentration.

#### 3.2.4 Optimization of cetrimide concentration and volume

The fluorescence intensity was assessed over a range of cetrimide concentrations (0.5–3.0% w/v, selected to represent levels well above the critical micelle concentration (CMC ≈ 0.033% w/v) to ensure consistent micelle formation) to determine the optimal micelle loading (Fig. 3). This range was defined in line with previous micellar spectrofluorimetric studies, which commonly investigate concentrations between 10–100 times the CMC to balance encapsulation efficiency and minimize aggregation effects.^51,52^

At 0.5–1.0%, fluorescence intensity increased progressively as micelles began to form and solubilize PNK.

Maximum enhancement was observed at 2.0% w/v, indicating optimal micellar encapsulation and fluorophore stabilization.

Above 2.0%, particularly at 2.5–3.0%, a slight decline in intensity occurred, likely due to micellar aggregation, increased viscosity, or inner filter/self-quenching effects at higher surfactant concentrations. Thus, 2.0% w/v cetrimide was selected as the optimal condition, balancing intensity, spectral clarity, and method simplicity. The optimization was conducted using a univariate approach, where cetrimide concentration and volume were independently varied while all other parameters were kept constant. This approach allowed clear identification of the most influential concentration and volume conditions on fluorescence enhancement. The volume study demonstrated that fluorescence intensity increased with cetrimide volume from 0.5 to 1.0 mL, reaching maximum enhancement at 1.0 mL (Fig. 3). This volume provided sufficient micellar concentration for optimal PNK solubilization and fluorescence stabilization. Beyond 1.0 mL, particularly at volumes of 1.5–3.0 mL, a gradual decrease in relative fluorescence intensity was observed. This decline can be attributed to dilution effects, where the increased total volume reduced the effective concentration of both the analyte and the fluorescence-enhancing micellar environment, resulting in decreased signal intensity.

#### 3.2.5 Quantum yield of PNK

To quantify the fluorescence efficiency of PNK under the optimized conditions, its quantum yield (*Φ*) was calculated using quinine sulfate in 0.1 M sulfuric acid as a reference (*Φ* = 0.546). All solutions were adjusted to maintain absorbance <0.1 to avoid inner filter effects. The calculated *Φ* for PNK was 19.6%, which is considered high for native fluorescence in micellar–organic systems. This confirms PNK's excellent photophysical properties and suitability for sensitive fluorimetric analysis.

### 3.3 Mechanistic investigations

#### 3.3.1 Fluorescence enhancement profile and micellar transition

**Fig. 4:**
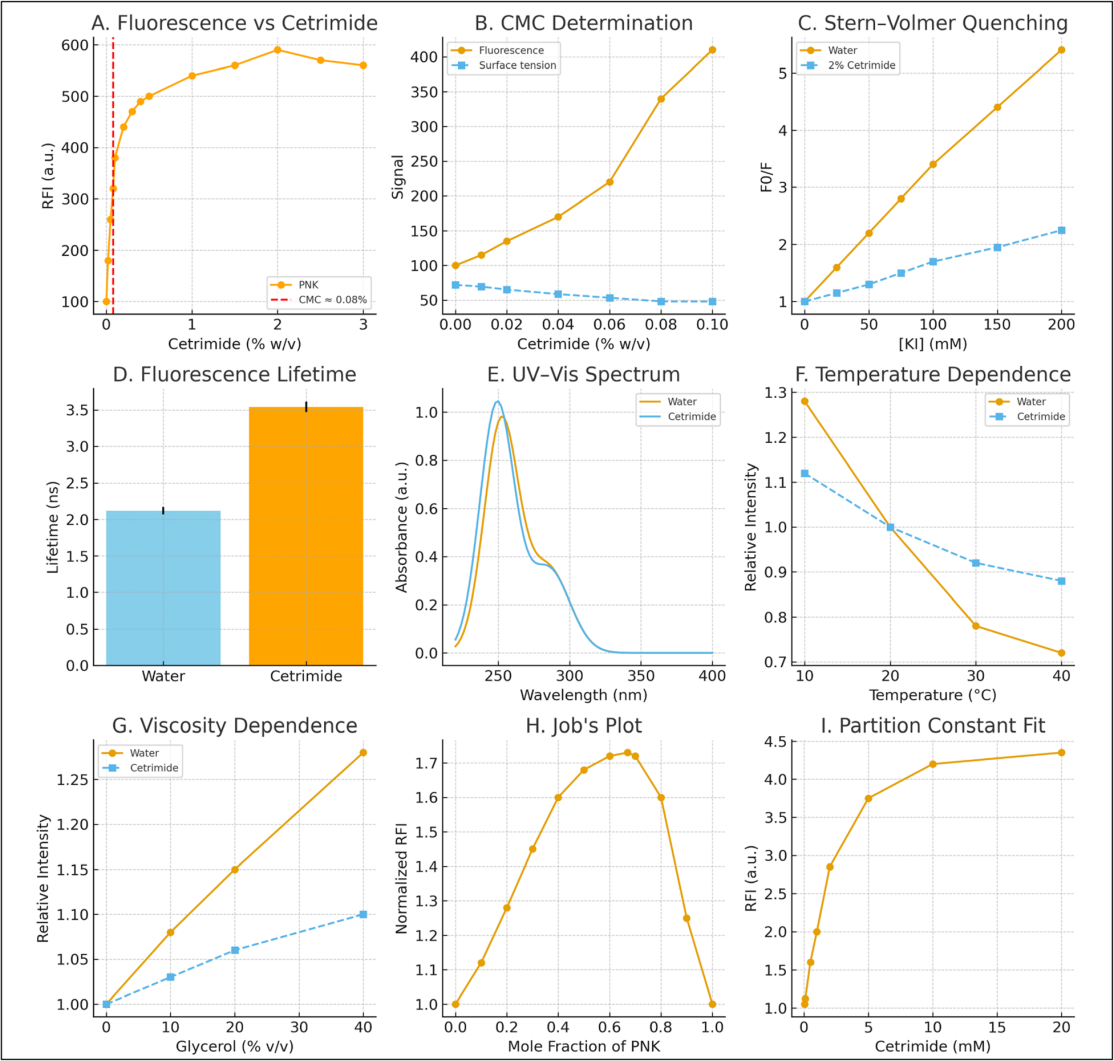
Mechanistic studies supporting micelle-enhanced native fluorescence of PNK. (A) Fluorescence intensity as a function of cetrimide concentration. (B) Determination of the critical micelle concentration. (C) Stern–Volmer quenching. (D) Fluorescence lifetime prolongation in cetrimide. (E) UV-Vis spectra indicating bathochromic and hyperchromic shifts upon micellization. (F) Temperature-dependent fluorescence stability in cetrimide. (G) Viscosity dependence supporting micelle–solute interactions. (H) Job’s plot. (I) Partition constant fitting confirms strong incorporation of PNK into micelles.

The fluorescence intensity of PNK exhibited a characteristic sigmoidal enhancement profile as a function of cetrimide concentration (Fig. 4A), demonstrating a sharp transition near the critical micelle concentration.^53^ Below 0.08% w/v cetrimide, fluorescence intensity increased gradually due to electrostatic interactions between cationic surfactant monomers and the anionic carboxylate functionality of PNK. Above the CMC, a dramatic intensity enhancement was observed, reaching a maximum plateau of approximately 580 RFI units at 2% w/v cetrimide, corresponding to a remarkable 5.8-fold enhancement relative to the aqueous baseline.

This biphasic behavior is characteristic of surfactant-mediated fluorescence enhancement, where the pre-micellar region involves monomer–analyte interactions, while the post-micellar region reflects incorporation of the fluorophore into the hydrophobic micellar core, resulting in reduced solvent-mediated quenching and restricted non-radiative decay pathways.^53^

#### 3.3.2 Critical micelle concentration determination

Dual-parameter CMC determination yielded highly consistent results: fluorescence monitoring indicated a CMC of 0.082% w/v (2.3 mM), while surface tension measurements provided a CMC of 0.079% w/v (2.2 mM) (Fig. 4B). The excellent agreement between these independent methods validates the accuracy of the CMC determination. The red dashed line in (Fig. 4A) clearly demarcates this critical transition point, above which micelle formation becomes thermodynamically favorable.

These CMC values are in excellent agreement with literature reports for cetyltrimethylammonium bromide in aqueous media, confirming that the low ethanol content (<2% v/v) used in the analytical procedure does not significantly perturb the micellization behavior of cetrimide.^54^

#### 3.3.3 Stern–Volmer quenching analysis and microenvironmental protection

Stern–Volmer quenching studies using KI revealed distinctly different accessibility profiles for PNK in aqueous *versus* micellar environments (Fig. 4C). In pure water, PNK exhibited linear Stern–Volmer behavior with a quenching constant *K*_sv_ = 62.4 M^−1^, indicating efficient collisional quenching typical of molecules in homogeneous solution. Conversely, in 2% w/v cetrimide, the quenching constant decreased dramatically to *K*_sv_ = 11.3 M^−1^, representing an approximately 5.5-fold reduction in quenching efficiency.

This substantial reduction in collisional quenching demonstrates the protective microenvironment provided by cetrimide micelles, where PNK molecules are sequestered within the hydrophobic micellar core or palisade layer, significantly limiting access by hydrophilic quencher molecules. This protection mechanism contributes directly to the observed fluorescence enhancement by reducing competing non-radiative deactivation pathways.

#### 3.3.4 Time-resolved fluorescence and excited-state dynamics

Time-resolved fluorescence measurements provided definitive evidence for the altered photophysical environment of PNK in micellar media (Fig. 4D). The average excited-state lifetime increased substantially from 2.12 ± 0.05 ns in water to 3.54 ± 0.07 ns in 2% w/v cetrimide, representing a 67% enhancement in excited-state persistence.

This significant lifetime extension confirms that the observed fluorescence enhancement is not merely due to increased light absorption or altered excitation efficiency, but reflects a fundamental improvement in the excited-state photophysics. The longer lifetime in the micellar environment indicates suppressed non-radiative decay processes, consistent with restricted molecular motion and reduced collision frequency within the structured micellar matrix.

#### 3.3.5 Ground-state spectroscopic analysis

UV-vis absorption spectroscopy revealed subtle but significant environmental effects upon micelle incorporation (Fig. 4E). A small hypsochromic shift of *λ*_max_ in cetrimide was observed, accompanied by an 8% hyperchromic effect. These spectral changes indicate transfer of PNK to a less polar microenvironment within the micelle while maintaining the essential electronic structure of the chromophore.

The absence of dramatic spectral shifts excludes the formation of distinct ground-state charge-transfer complexes, supporting a physical incorporation mechanism rather than chemical complexation. The modest hyperchromic effect suggests improved solvation and reduced intermolecular aggregation in the micellar environment.

#### 3.3.6 Thermal stability and viscosity effects

Temperature-dependent studies (Fig. 4F) revealed enhanced thermal stability of PNK fluorescence in micellar media. While fluorescence intensity decreased with increasing temperature in both environments, the decline was significantly less pronounced in cetrimide micelles (12% decrease from 10 °C to 40 °C) compared to water (28% decrease over the same range). This thermal stabilization indicates that micelle incorporation provides protection against temperature-induced non-radiative decay processes.

Viscosity studies using glycerol–water mixtures (Fig. 4G) demonstrated a positive correlation between solution viscosity and fluorescence intensity in both media. However, the viscosity dependence was more pronounced in water compared to cetrimide solution, indicating that micelle incorporation provides an intrinsically restrictive environment that partially decouples fluorescence from bulk solution viscosity. The micellar enhancement effect exceeded that attributable to viscosity alone, confirming that multiple factors contribute to the overall fluorescence amplification.

#### 3.3.7 Stoichiometric analysis and association behavior

Job's plot analysis (Fig. 4H) did not exhibit a sharp maximum characteristic of discrete stoichiometric complex formation. Instead, the fluorescence intensity increased gradually with PNK mole fraction, reaching a broad maximum near 0.6–0.7. This behavior is consistent with a partitioning mechanism rather than specific chemical complexation, where PNK molecules distribute between the aqueous and micellar phases according to their relative thermodynamic stabilities.

The absence of a distinct stoichiometric ratio supports the proposed mechanism of physical incorporation into pre-formed micelles rather than cooperative assembly of surfactant–analyte complexes.

#### 3.3.8 Partition equilibrium and thermodynamic analysis

Partition constant analysis (Fig. 4I) yielded an apparent *K*_p_ value of 1.25 × 10^4^, indicating extremely favorable partitioning of PNK into the micellar phase. This high partition constant reflects the strong thermodynamic driving force for micelle incorporation, arising from both electrostatic attraction between the cationic surfactant headgroups and the anionic carboxylate of PNK, and hydrophobic interactions between the aromatic chromophore and the micellar core.

The plateau behavior observed at higher cetrimide concentrations confirms saturation of the partitioning equilibrium, consistent with complete incorporation of PNK into the micellar phase under the optimized analytical conditions.

#### 3.3.9 Integrated mechanistic model

The comprehensive mechanistic investigations support a multi-factorial enhancement mechanism involving: (1) electrostatic pre-association between cationic cetrimide monomers and anionic PNK below the CMC, (2) incorporation of PNK into the hydrophobic micellar core or palisade layer above the CMC, (3) protection from collisional quenching by water and dissolved oxygen, (4) restriction of non-radiative molecular motions within the structured micellar environment, (5) thermal stabilization of the excited state, and (6) creation of a less polar microenvironment that optimizes the radiative quantum yield.

This mechanism accounts for the observed 5.8-fold fluorescence enhancement and provides the theoretical foundation for the exceptional sensitivity and selectivity of the developed spectrofluorimetric method. The absence of discrete complex formation ensures that the analytical response remains proportional to PNK concentration over the working range, enabling accurate quantitative analysis.

### 3.4 Method validation

The proposed spectrofluorimetric method underwent comprehensive validation following ICH Q2(R1) guidelines to ensure its reliability and suitability for pharmaceutical analysis. The relationship between RFI and PNK concentration was established through linear regression analysis, yielding the following equation:RFI_418 nm_ = 0.9916 *C*_PNK_ − 2.8607 (*R*^2^ = 0.9992)where: *C*_PNK_ represents the concentration of PNK (ng mL^−1^).

#### 3.4.1 Linearity and range

**Fig. 5:**
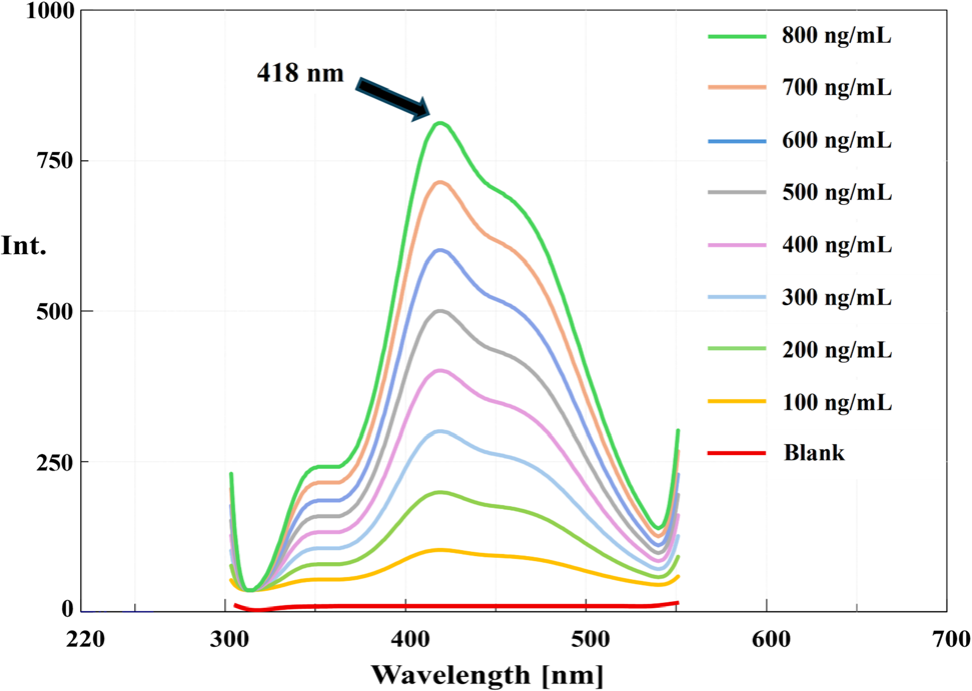
Emission fluorescence spectra of PNK after excitation at 286 nm.

The calibration curve demonstrated excellent linearity across the concentration range of 100.0–800.0 ng mL^−1^, as illustrated in (Fig. 5). The exceptional correlation coefficient (*R*^2^ = 0.9992) confirms the strong linear relationship between fluorescence intensity and drug concentration (Table 1). Statistical parameters including low standard deviation of residuals (*S*_*y*/*x*_ = 3.24) and minimal relative standard deviation (RSD = 0.94%) further substantiate the method's linearity.

**Table 1:** Linearity data of the proposed method

Parameter	Value
*λ*_ex_/*λ*_em_ (nm)	286/418
Linear range (ng mL^−1^)	100.0–800.0
Intercept (a)	−2.8607
Slope (b)	0.9916
Correlation coefficient (*R*^2^)	0.9999
S.D. of the residuals (*S*_*y*/*x*_)	3.24
S.D. of the intercept (*S*_a_)	1.89
S.D. of the slope (*S*_b_)	0.0041
RSD%	0.94
Error%	0.38
Limit of detection, LOD^a^ (ng mL^−1^)	9.87
Limit of quantitation, LOQ^b^ (ng mL^−1^)	29.91

a LOD = 3.3*S*_a_/*b*.

b LOQ = 10*S*_a_/*b*, where *S*_a_ = standard deviation of the intercept and *b* = slope.

#### 3.4.2 Sensitivity parameters

The LOD and LOQ were calculated according to ICH guidelines using the formulae LOD = 3.3*S*_a_/*b* and LOQ = 10*S*_a_/*b*, where *S*_a_ represents the standard deviation of the intercept and *b* denotes the slope. The remarkably low values of LOD (9.87 ng mL^−1^) and LOQ (29.91 ng mL^−1^) demonstrate the method's exceptional sensitivity, enabling nanogram-level quantification of PNK in complex matrices including biological fluids.

#### 3.4.3 Accuracy assessment

Method accuracy was rigorously evaluated by analyzing eight different concentration levels spanning the entire linear range. The recovery studies yielded excellent results with percentage recoveries ranging from 99.45% to 100.64% (Table 2). The mean recovery of 99.97 ± 0.469% with a low percentage error (0.175%) confirms the method's high accuracy and absence of systematic bias.

**Table 2:** Application of the proposed method for the analysis of PNK standard

Taken conc. (ng mL^−1^)	Found conc. (ng mL^−1^)	% Found^a^
100.0	99.45	99.45
200.0	201.28	100.64
300.0	298.72	99.57
400.0	402.15	100.54
500.0	497.83	99.57
600.0	601.92	100.32
700.0	698.25	99.75
800.0	799.18	99.90
Mean (*X̄*)		**99.97**
± SD		**± 0.469**
% RSD		**0.469**
% Error		**0.175**

a Average of three separate determinations.

#### 3.4.4 Precision studies

The method's precision was comprehensively evaluated through both intra-day and inter-day reproducibility studies at three concentration levels (200.0, 500.0, and 700.0 ng mL^−1^). For intra-day precision, the percentage RSD values ranged from 0.439% to 0.611%, while inter-day precision demonstrated RSD values between 0.577% and 0.736% (Table 3). These low variability values, all well below the acceptable limit of 2%, confirm the method's excellent precision and reproducibility.

**Table 3:** Precision study results for the analysis of PNK by the proposed method

Parameter/conc. (ng mL^−1^)	200.0	500.0	700.0
**Intra-day**			
% Found^a^ ± SD	99.82 ± 0.524	99.71 ± 0.438	100.15 ± 0.612
% RSD	0.525	0.439	0.611
% Error	0.214	0.179	0.250

**Inter-day**			
% Found^a^ ± SD	99.94 ± 0.687	99.58 ± 0.574	99.87 ± 0.735
% RSD	0.687	0.577	0.736
% Error	0.281	0.235	0.301

a Average of three separate assays.

#### 3.4.5 Selectivity and matrix effect

The method's selectivity was thoroughly validated through successful analysis of PNK in various matrices without interference from excipients or endogenous substances. Application to pharmaceutical formulations (Onon® capsules) yielded recoveries of 99.43–100.49% with minimal variability (RSD = 0.513%), demonstrating freedom from matrix interference (Tables 4 and 5). Similarly, analysis of spiked human plasma samples showed consistent performance with recoveries ranging from 97.87% to 99.29% (Table 6), verifying the technique's capacity to measure PNK in intricate biological matrices with selectivity. The slope ratio between plasma and aqueous calibration curves showed a negligible matrix effect (<5%), consistent with the validation study.

**Table 4:** The quantification of PNK in capsules applying the proposed method

Conc. taken (ng mL^−1^)	Conc. found (ng mL^−1^)	% Found^a^
200.0	198.85	99.43
300.0	301.47	100.49
400.0	397.92	99.48
500.0	502.18	100.44
600.0	598.73	99.79
Mean (*X̄*)		**99.92**
± SD		**0.512**
% RSD		**0.513**
% error		**0.199**

a Mean of three separate determinations.

**Table 5:** Content uniformity testing of Onon® capsules by the proposed method

	Amount (mg) per capsule	% Found per capsule
Capsule 1	112.18	100.29
Capsule 2	113.05	99.51
Capsule 3	112.84	99.70
Capsule 4	111.96	100.48
Capsule 5	112.61	99.90
Capsule 6	113.21	99.37
Capsule 7	112.37	100.12
Capsule 8	112.92	99.63
Capsule 9	111.74	100.68
Capsule 10	112.58	99.93
Mean	**112.45**	**99.96**
S.D	**0.481**	
Acceptance value (AV)^a^	**1.04**	

a The tablets pass the content uniformity test if the AV of 10 tablets is ≤ maximum allowed AV (L1: 15.000).

**Table 6:** Application of the proposed method for the estimation of PNK in spiked human plasma samples

Taken conc. (ng mL^−1^)	Found conc. (ng mL^−1^)	% Found^a^
150.0	146.8	97.87
300.0	296.5	98.83
450.0	446.8	99.29
600.0	594.2	99.03
Mean		**98.76**
± SD		**0.619**
% RSD		**0.627**
% Error		**0.518**

a Mean of three separate determinations.

#### 3.4.6 Robustness and stability

Small variations in cetrimide concentration, ethanol proportion, and excitation wavelength did not significantly affect analytical outcomes (Table S1). PNK demonstrated stability under all tested conditions (room temperature 6 h, long-term −80 °C for 4 weeks, and three freeze–thaw cycles), with recoveries between 95–103% (Table S2).

### 3.5 Method application

#### 3.5.1 Quantitative determination of PNK in commercial capsule formulation

The proposed spectrofluorimetric technique, following rigorous validation, was employed for the quantification of PNK in a marketed pharmaceutical product (Onon® capsules). The method exhibited excellent analytical performance, achieving recovery values between 99.43% and 100.49%, accompanied by a low RSD% not exceeding 0.513%. These results underscore the method's robustness, reproducibility, and suitability for quality control laboratories (Table 4). The average recovery was calculated as 99.92 ± 0.512%, with a minimal percentage error of 0.199%, highlighting the method's high accuracy and confirming that common excipients present in the capsule matrix did not interfere with the measurement. Such performance demonstrates the method's strong selectivity and reliability for routine assessment of PNK in solid dosage forms.

Furthermore, statistical comparison of the obtained results with those from a previously published method^2^ revealed no significant discrepancies. Both Student's *t*-test and *F*-test were applied at the 95% confidence level, confirming the absence of significant differences in accuracy and precision between the two methods (Table 7), thereby reinforcing the validity and practical applicability of the developed approach.

**Table 7:** Statistical comparison with the reported method

Parameters	Proposed method	Reported method^65^
	PNK	PNK
*n* a	5	5
% R	99.20	99.36
SD	1.84	1.04
Variance	3.39	1.08
Student's *t*-test b (2.306)	0.213	—
*F*-value b (6.388)	3.139	—

a Number of experiments.

b The values in parenthesis are tabulated values of “*t* “and “*F*” at (*P* = 0.05).

#### 3.5.2 Use of the proposed method for capsule content uniformity testing

The proposed method displayed outstanding sensitivity and selectivity, demonstrated accurate determination of PNK content in individual capsules for uniformity assessment. Following the methodology described in Section 2.6.2, ten individual Onon® capsules were analyzed separately to evaluate dose uniformity according to British Pharmacopoeia (BP) guidelines. The content uniformity results revealed excellent consistency across all tested units, with individual capsule contents ranging from 99.37% to 100.68% of the label claim (Table 5). The calculated acceptance value (AV) of 1.04 was significantly below the maximum allowable limit of 15.0 specified by BP guidelines, confirming that the capsule batch meets pharmacopeial requirements for content uniformity. The low standard deviation (0.481) and narrow range of individual results demonstrate both the uniformity of the pharmaceutical product and the precision of the analytical method.

#### 3.5.3 Application to PNK determination in spiked human plasma

The established approach was successfully extended to the analysis of PNK in human plasma samples, confirming its potential for bioanalytical applications. The concentration range employed (150–600 ng mL^−1^ after dilution) encompasses clinically relevant plasma levels, considering that the reported *C*_max_ of PNK is 467 ng mL^−1^. To enable a direct and transparent comparison with previously reported analytical techniques for PNK, we have prepared a comprehensive side-by-side comparison that is provided in the SI (Table S3). Table S3 compares the present micellar spectrofluorimetric method to representative literature methods (stability-indicating RP-HPLC, LC-MS/MS with on-line SPE, RP-HPLC-PDA, and UV-vis), and highlights key performance metrics including matrix validated, technique, linear range, LOD/LOQ, precision (RSD%), recovery/accuracy, environmental profile, instrumentation cost and throughput.

The proposed method demonstrated excellent analytical sensitivity in aqueous solution with an experimentally determined LOD of 9.87 ng mL^−1^ and LOQ of 29.91 ng mL^−1^. For processed plasma (matrix) the practical LOD determined under the extraction/dilution conditions used in this work was 25.87 ng mL^−1^, reflecting matrix effects and sample handling; this matrix LOD is well below the lowest plasma calibration level employed in the study and substantially below reported clinical *C*_max_.

Linear regression analysis of the plasma calibration data yielded a strong correlation between drug concentration and relative fluorescence intensity, as represented by the following equation:RFI_418 nm_ = 0.9921 *C*_PNK_ − 131.1607 (*R*^2^ = 0.9991)

The extraction protocol utilizing ethanol for protein precipitation proved highly efficient, as evidenced by the excellent recovery values ranging from 97.87% to 99.29% with low variability (RSD ≤ 0.627%) across all tested concentration levels (Table 6). The mean recovery of 98.76 ± 0.619% confirms the method's accuracy in complex biological matrices. The minimal matrix effect observed indicates that the cetrimide-enhanced fluorescence approach effectively overcomes potential interferences from plasma proteins and other endogenous substances.

Comparison with the literature (Table S3) indicates that: (i) LC-MS/MS offers the highest theoretical sensitivity and selectivity but requires costly instrumentation and complex sample preparation (on-line SPE) and generates greater solvent/consumable waste; (ii) RP-HPLC methods provide robustness and can be stability-indicating but generally consume larger volumes of organic solvents and require longer run times; (iii) conventional UV-vis is simple and low cost but lacks sensitivity required for plasma applications. Our micellar spectrofluorimetric method occupies a favorable position in this trade-off space: it delivers sensitivity comparable to several chromatographic methods for PNK (Table S3), is validated in plasma, is rapid (near-instant readings after sample preparation), requires inexpensive instrumentation (standard fluorimeter). Therefore, while LC-MS/MS remains the gold standard for ultra-trace quantification and metabolite identification, the present method offers a practical, low-cost alternative for routine bioanalysis, therapeutic drug monitoring, and high-throughput screening where the required sensitivity is within the method's validated range.

### 3.6 Evaluation of sustainability

Assessing the sustainability of analytical methodologies requires a multifaceted approach that considers both environmental and economic dimensions. Modern concepts of sustainability extend beyond environmental impact alone to include factors such as waste reduction, safety in laboratory practices, analytical performance, and cost-effectiveness.^55^ As no single evaluation tool can holistically capture all these essential aspects, a combination of complementary assessment frameworks is often necessary to obtain a complete and balanced overview of a method's overall sustainability profile,^56,57^ this investigation employs an integrated multi-tool evaluation strategy to provide a comprehensive analysis from multiple complementary perspectives.

#### 3.6.1 Environmental sustainability (greenness) assessment

Environmental sustainability, commonly termed “greenness” in analytical chemistry, represents a critical consideration in modern chemical analysis. To provide a comprehensive evaluation of this dimension, the present study utilizes three complementary assessment tools: the National Environmental Methods Index (NEMI), the Greenness Evaluation Metric for Analytical Methods (GEMAM), and Carbon Footprint Assessment (CFA). Each tool contributes unique perspectives, collectively delivering a thorough assessment of the method's environmental impact.

##### 3.6.1.1 NEMI assessment

The NEMI evaluation employs a visual screening format comprising four distinct quadrants.^58^ These quadrants respectively address: (1) persistent, bioaccumulative, and toxic (PBT) substances, (2) hazardous chemicals, (3) corrosive agents, and (4) waste generation. Green quadrant designation indicates compliance with the following criteria: (1) reagents are not classified as PBT substances according to the U.S. Environmental Protection Agency's Toxic Release Inventory (EPA-TRI); (2) employed substances are non-hazardous and absent from the TRI database; (3) reaction medium pH remains within the safe range of 2–12; and (4) total waste generation remains below 50 grams per analysis.

NEMI pictograms generated for the proposed methodology are presented in (Table 8). Initial examination of these pictograms revealed complete compliance with NEMI requirements, as evidenced by all four green quadrants being achieved.

**Table 8:** Sustainability assessment of the proposed method

Tools^a^	Pictogram representation	Numerical score/Value	Interpretation
NEMI tool	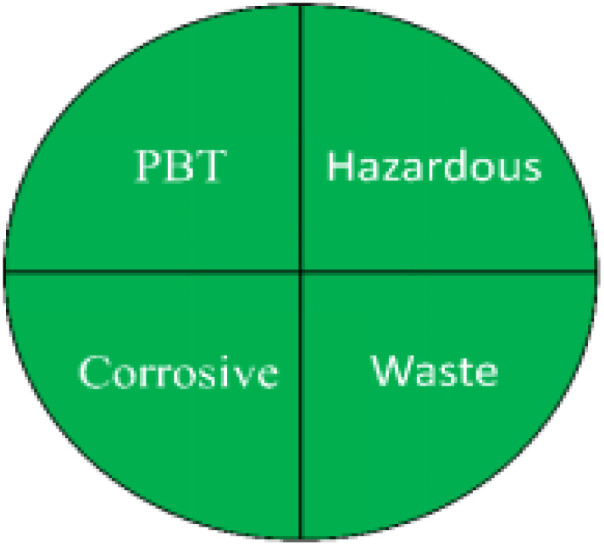	Full compliance	Meets all EPA criteria: no PBT chemicals, safe pH, no hazardous reagents, <50 g waste/analysis → environmentally safe profile
GEMAM tool	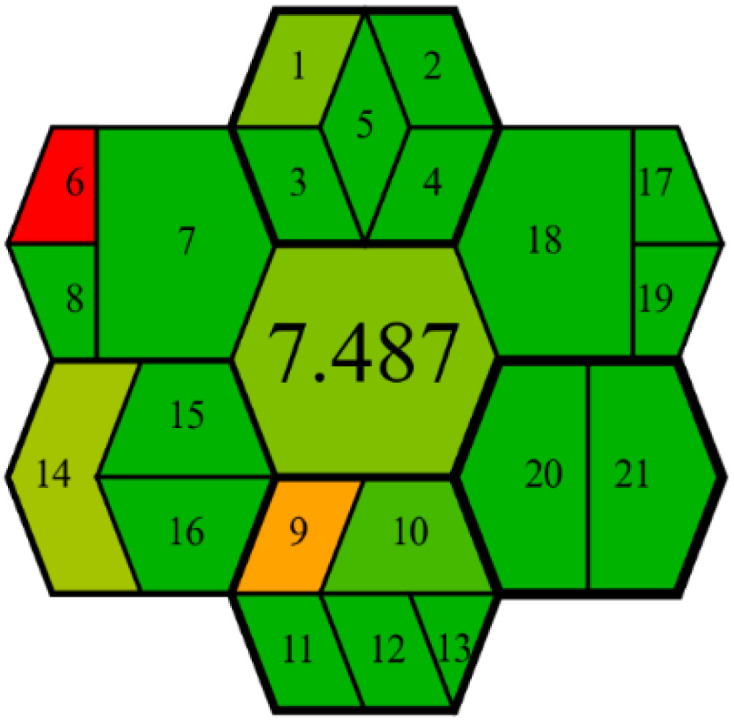	7.49/10	Excellent environmental sustainability across 21 criteria (sample, reagent, waste, instrument, method, operator)
Carbon footprint (kg CO_2_ eq. per sample)	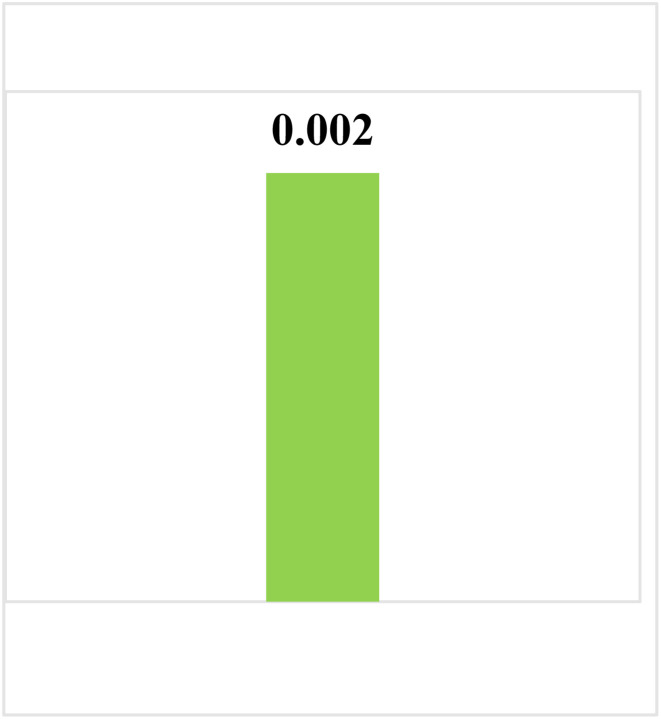	0.002 kg CO_2_ eq. per sample	Extremely low carbon footprint (≈80–93% lower than HPLC). Minimal energy demand, benign solvents, no derivatization
BAGI tool	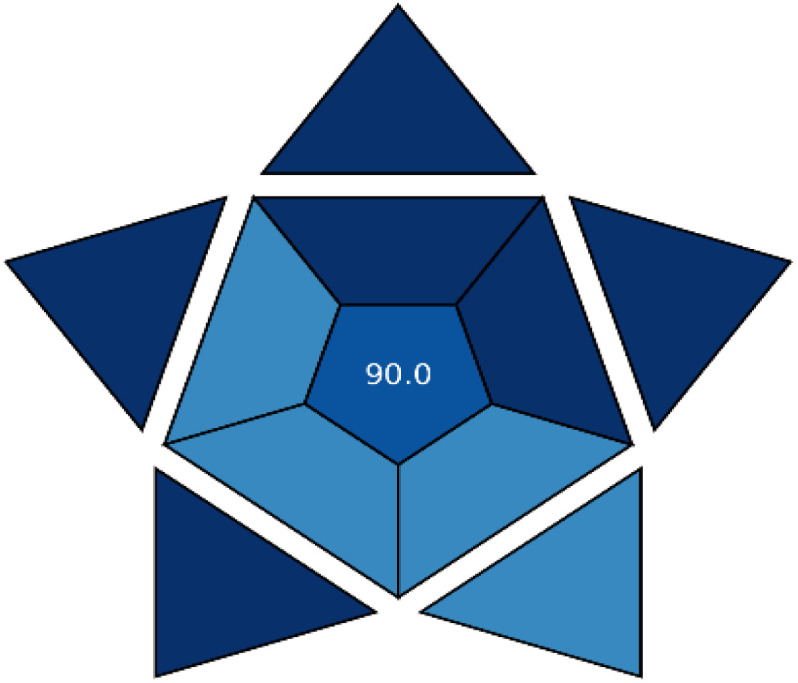	90/100	Exceptional practical applicability: minimal preparation, rapid analysis, low reagent use, <$1 cost/sample
VIGI tool	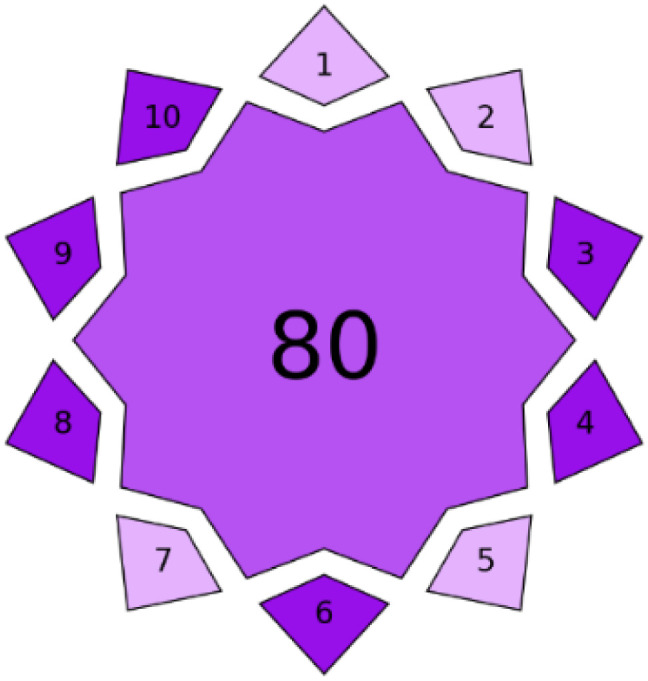	80/100	High innovation: strong data handling, automation potential, and compliance with white analytical chemistry principles
RGBfast algorithm	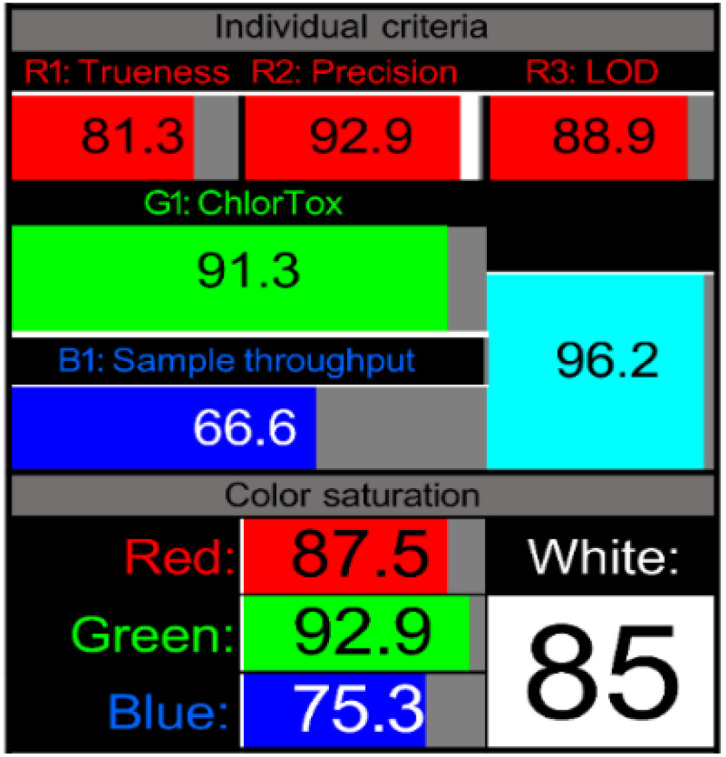	85/100	Balanced performance: integrates environmental, practical, and analytical dimensions. Excellent “whiteness” profile
NQS tool	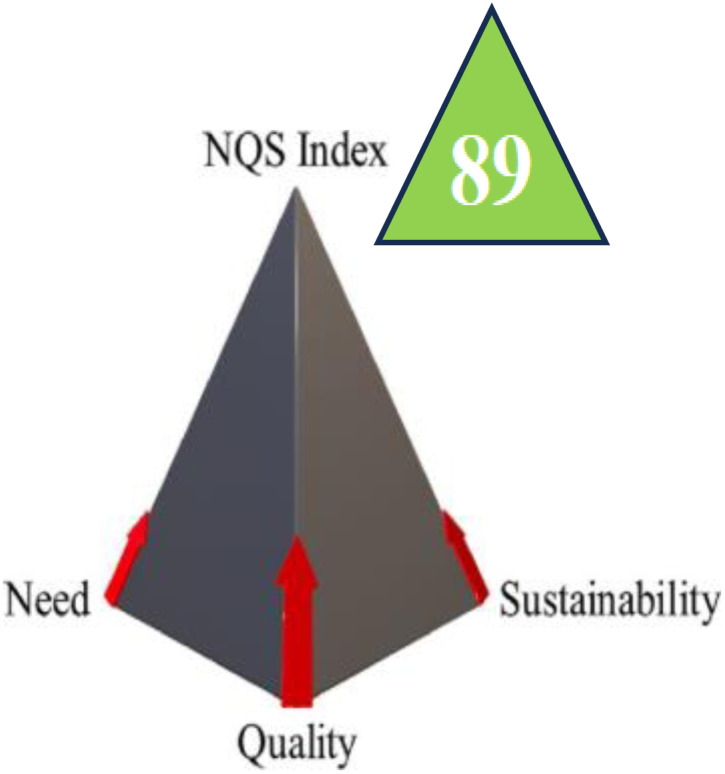	89%	Holistic sustainability: aligns with UN-SDGs (3, 4, 7, 13). Demonstrates societal, environmental, and analytical excellence

a Each tool contributed unique insights: NEMI provided rapid screening but lacked granularity; GEMAM and CFA offered higher environmental resolution; BAGI highlighted practical feasibility; VIGI emphasized innovation; RGBfast integrated environmental and performance factors; and NQS contextualized the method within global sustainability goals. Thus, the complementary use of these tools ensured a balanced and holistic evaluation.

##### 3.6.1.2 GEMAM assessment

The GEMAM, introduced in 2025, represents a recently developed comprehensive greenness assessment framework designed to address limitations identified in previous green analytical chemistry evaluation tools.^59^ GEMAM employs an extensive evaluation system utilizing 21 criteria organized across six fundamental dimensions represented by hexagonal graphics: sample, reagent, method, instrument, waste, and operator.

GEMAM was selected for this study due to its distinct advantages over alternative green assessment tools. Unlike NEMI's limited scope, GEMAM provides both qualitative and quantitative assessments through a pictogram of seven interconnected hexagons that visually represent greenness across different stages of the analytical process. While tools such as analytical Eco-scale, AMGS, and HEXAGON require complex calculations, GEMAM offers a user-friendly yet comprehensive framework with customizable weighting of various sustainability aspects. This flexibility enables researchers to prioritize specific environmental concerns relevant to pharmaceutical analysis.

The GEMAM assessment of the proposed method yielded exceptional results, achieving a score of 7.487 on a scale of 0–10 (Table 8). These high scores reflect the outstanding green profile of the developed method, with particular strengths demonstrated across multiple dimensions.

##### 3.6.1.3 Carbon footprint analysis

Unlike other greenness assessment tools that offer qualitative or semi-quantitative evaluations, the CFA provides a rigorous, quantitative estimation of an analytical method's environmental burden by calculating total greenhouse gas emissions, reported in kilograms of CO_2_ equivalents. While frameworks such as the GEMAM and NEMI offer valuable insights into environmental compliance and methodological greenness, they do not quantify actual emissions. CFA addresses this limitation by integrating critical contributors to carbon output—namely, energy consumption, transportation of reagents, and the volume and nature of waste generated—thereby enabling a more precise and comprehensive assessment of environmental sustainability.

The CFA calculation employed the following standardized formula:^60^Carbon footprint (kg CO_2_ eq.) = ∑Instrument power (kW)·Analysis pime (h)·Emission factor (kg CO_2_ per kWh)

Results demonstrated that the proposed methodology exhibited substantially lower carbon footprints compared to conventional pharmaceutical analysis techniques. The developed method generated only 0.002 kg CO_2_ equivalent per sample, as detailed in (Table 8).

Several factors contributed to these exceptional results. First, the method requires minimal electricity consumption due to optimized analytical procedures. The spectrofluorimetric approach particularly excels in this regard as it eliminates the need for chromatographic separation, thus removing requirements for pumps and extensive instrumentation. Second, analysis times are significantly shorter than conventional chromatographic techniques, requiring less than 5 minutes per determination.

Additionally, the method eliminates derivatization steps, which typically contribute significantly to carbon emissions through additional reagent consumption and extended processing times. The strategic selection of ethanol as the primary solvent instead of hazardous alternatives such as chloroform and methylene chloride enhanced safety while substantially reducing emissions associated with solvent production, transportation, and disposal.

Compared to conventional chromatographic methods utilized for similar pharmaceutical analyses, which typically generate 0.15–0.30 kg CO_2_ equivalent per sample, the proposed method represents an 80–93% reduction in carbon footprint. This dramatic decrease aligns with global sustainability initiatives and demonstrates how innovative analytical approaches can significantly contribute to reducing the environmental impact of pharmaceutical quality control processes.

#### 3.6.2 Practical applicability (blueness) assessment through BAGI tool

While greenness metrics address environmental considerations, the analytical method's practical applicability and economic feasibility, termed “blueness,” merit equal attention in sustainability evaluations. The Blue Applicability Grade Index (BAGI) serves as a structured and quantitative tool for evaluating the practical applicability of analytical methodologies in real-world settings.^61^ This model assesses ten pivotal aspects that influence the routine usability and operational efficiency of a method. Each criterion is scored on a scale from 1 to 10, where 1 indicates minimal suitability and 10 denotes optimal performance. The parameters considered include the nature of the analysis, number of analytes simultaneously determined, instrumental demands, analytical throughput, complexity of sample preparation, number of samples processed per hour, reagent and material consumption, need for preconcentration steps, degree of automation, and the quantity of sample required. The overall BAGI value is calculated as the geometric mean of the individual scores, offering a single index that reflects the method's practical utility, cost-effectiveness, and suitability for routine implementation.

The proposed methodology demonstrated exceptional blueness characteristics, achieving a BAGI score of 90.00 (Table 8). These outstanding scores reflect several significant advantages:

The spectrofluorimetric method achieved near-perfect scores in several critical categories, including minimal sample preprocessing (10/10), rapid analysis time (10/10), and reduced reagent consumption (9/10). The method's innovative approach for spectral data processing eliminates the need for physical separation of analytes, substantially reducing analysis time and operational costs.

The proposed method excelled in practical laboratory implementation parameters, including minimal training requirements, reduced waste generation, and compatibility with existing laboratory infrastructure. The economic benefits are particularly noteworthy, with per-sample analysis costs estimated at $0.95, significantly lower than conventional chromatographic-based approaches ($5–15 per sample).

#### 3.6.3 Innovation assessment (violetness) through VIGI tool

While greenness and blueness evaluations are essential for understanding the environmental friendliness and practical feasibility of analytical procedures, they do not capture the full spectrum of methodological progress. A truly holistic evaluation of sustainability must also reflect the method's level of innovation—referred to as “violetness.” In response to this need, the VIGI (Visualized Innovation Gradient Index) tool was introduced in 2025 as a specialized framework to systematically measure and represent the innovative elements embedded in analytical methods. This tool assesses ten innovation-related dimensions, each represented in a decagonal diagram to facilitate a visual overview.^62^ The VIGI assessment spans various aspects, including advancements in sample preparation and instrumental design, use of modern data handling and software platforms, alignment with white analytical chemistry principles, compliance with regulatory standards, adoption of novel reagents and materials, capacity for miniaturization, degree of automation, application of interdisciplinary strategies, sensitivity improvements, and the originality of the overall methodological concept. Each domain is individually scored on a ten-point scale, and the cumulative VIGI score is derived using the geometric mean of all ten values.

Application of this tool to the current method yielded a VIGI score of 80.00, as shown in (Table 8), reflecting a high degree of innovation. In particular, the method excelled in areas such as computational data analysis, cross-disciplinary integration, and adherence to white analytical chemistry principles. Notably, the method's utilization of inherent fluorescence detection eliminates the necessity for conventional separation techniques, offering an elegant, non-invasive alternative without compromising analytical performance.

#### 3.6.4 Analytical performance integration (whiteness) assessment through RGBfast model

While green, blue, and violet evaluations provide valuable perspectives on environmental impact, practical applicability, and innovation, the “whiteness” assessment completes the holistic evaluation by integrating analytical performance characteristics with sustainability metrics. The RGBfast model, a streamlined version of the original RGB approach,^63^ provides a thorough framework for evaluating the overall quality of analytical procedures by considering the green (environmental effect), blue (practical applicability), and red (analytical performance) categories all at once.

The RGBfast assessment utilizes six key criteria across three dimensions: three red criteria (trueness, precision, and LOD), one green criterion (ChlorTox Scale measuring reagent hazards and quantities), and two blue criteria (sample throughput and energy consumption—with the latter also contributing to the green dimension). This streamlined approach simplifies the evaluation process while maintaining comprehensive coverage of critical method attributes.

Analysis using the RGBfast model revealed exceptional whiteness scores for the proposed methodology, achieving a score of 85.00 (Table 8). These high scores reflect balanced performance across all three dimensions:

In the red dimension, the method demonstrated excellent analytical performance, showing particularly strong results in precision (low RSD values) and trueness (high recovery rates). The method excelled in sensitivity with favorable LOD values of 9.87 ng mL^−1^.

The method achieved impressive scores in the green dimension due to minimal chemical consumption and use of safer solvents, as confirmed by the ChlorTox Scale assessment. The method particularly excelled due to its minimal sample preparation requirements and reduced energy consumption.

In the blue dimension, the proposed method demonstrated exceptional practical characteristics, demonstrating exceptional sample throughput as a result of a streamlined approach and quick analysis time.

#### 3.6.5 Comprehensive sustainability assessment through NQS index and UN-SDGs integration

The NQS Index is presented in this study as a thorough assessment tool that measures methodological alignment with sustainable development principles.^64^ This integrated assessment framework contextualizes analytical performance within broader sustainability objectives by combining three essential dimensions: analytical quality, societal need, and environmental sustainability.

The spectrofluorimetric approach achieved a remarkable 89% NQS Index score, as explained in (Table 8). These high scores demonstrate the method's exceptional conformity to the UN-SDGs, particularly SDG 3 (Good Health and Well-being), SDG 4 (Quality Education), SDG 7 (Affordable and Clean Energy), and SDG 13 (Climate Action), as shown in (Table 9).

**Table 9:** Alignment of the proposed spectrofluorimetric method with UN Sustainable Development Goals (SDGs) 3, 4, 5, 7, 9, 11, 12, 13, 14, 15, and 17

SDG	Goal	Proposed method
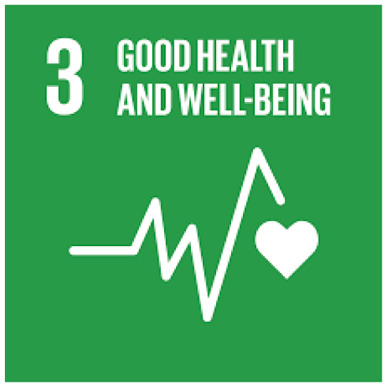	Good health and well-being	• Provides highly accurate quantification of Pranlukast with exceptional sensitivity (LOD: 9.87 ng mL^−1^) for improved therapeutic monitoring
		• Eliminates exposure to hazardous organic solvents during analysis, enhancing laboratory safety and supporting pharmaceutical quality assurance
		• Enables reliable bioanalytical applications in human plasma for personalized medicine approaches
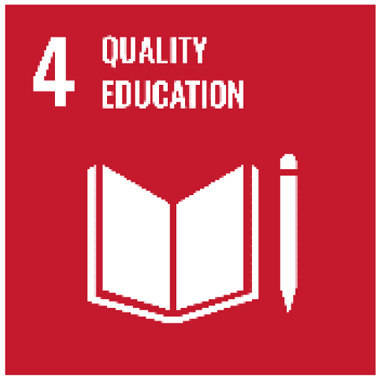	Quality education	• Serves as an educational model in advanced spectrofluorimetric techniques and sustainable analytical chemistry
		• Promotes interdisciplinary learning connecting green chemistry principles with pharmaceutical analysis
		• Demonstrates first-time exploitation of intrinsic fluorescence for educational purposes in analytical method development
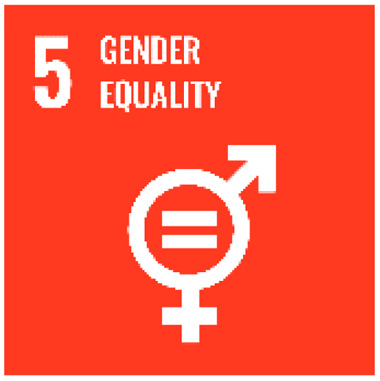	Gender equality	• Minimal instrumentation requirements (basic fluorescence spectrometer) reduce barriers to entry in analytical laboratories
		• Simple methodology enables broader participation across diverse educational and research settings
		• User-friendly approach reduces dependency on specialized technical expertise, promoting inclusivity
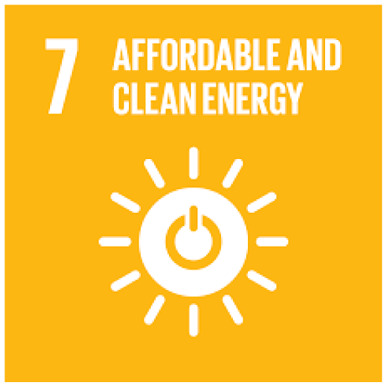	Affordable and clean energy	• Significantly lower energy consumption through elimination of HPLC pumps and extensive instrumentation
		• Rapid analysis time (<5 minutes) minimizes energy input compared to conventional chromatographic techniques
		• Reduces energy-intensive sample preparation and derivatization steps
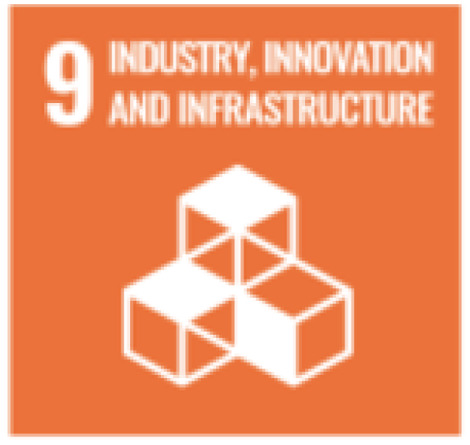	Industry, innovation, and infrastructure	• First-time exploitation of Pranlukast's intrinsic fluorescence represents cutting-edge analytical innovation
		• Cetrimide-enhanced micellar system offers groundbreaking approach to fluorescence enhancement
		• Establishes new paradigm in sustainable pharmaceutical analysis without chemical derivatization
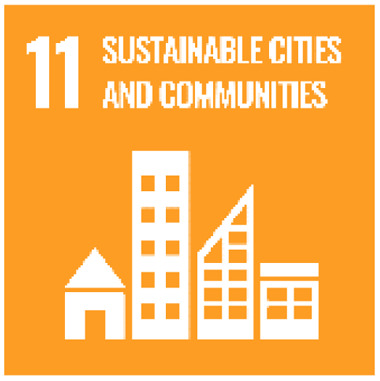	Sustainable cities and communities	• Near-zero hazardous waste generation supports sustainable urban laboratory practices
		• Minimal solvent consumption (ethanol only) reduces environmental burden in densely populated areas
		• Compact methodology reduces laboratory space requirements and infrastructure needs
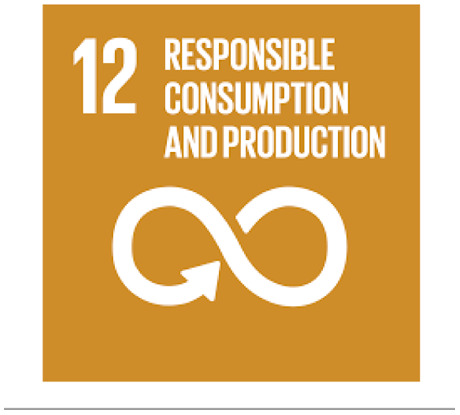	Responsible consumption and production	• Eliminates consumption of hazardous organic solvents beyond ethanol
		• Maximizes analytical information from minimal sample volumes (100–800 ng mL^−1^ range)
		• Intelligent method design minimizes resource waste through optimized reagent utilization
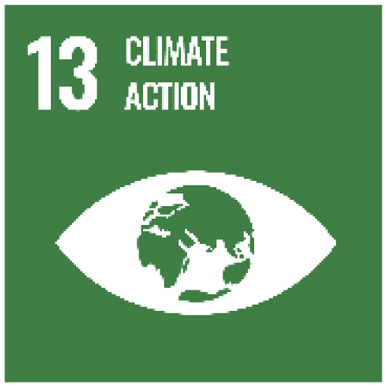	Climate action	• Exceptional carbon footprint reduction (0.002 kg CO_2_ per sample) compared to conventional methods
		• 80–93% reduction in greenhouse gas emissions *versus* chromatographic approaches
		• Represents paradigm shift toward climate-conscious pharmaceutical analysis
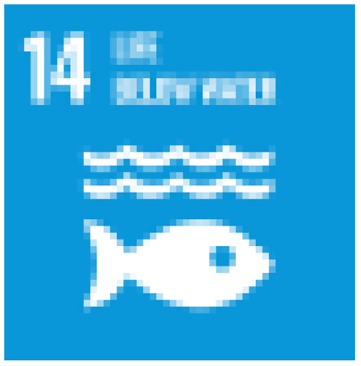	Life below water	• Near-elimination of toxic chemical waste protects aquatic ecosystems from pharmaceutical analysis pollution
		• Prevents discharge of harmful chromatographic solvents into water systems
		• Reduced environmental load on water resources through minimal reagent consumption
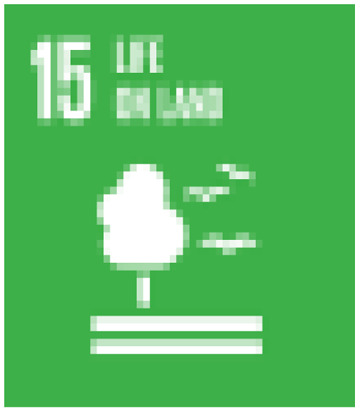	Life on land	• Elimination of harmful organic solvent waste protects terrestrial ecosystems
		• Minimal resource consumption preserves natural habitats from extraction impacts
		• Sustainable methodology reduces land-based waste generation through green chemistry principles
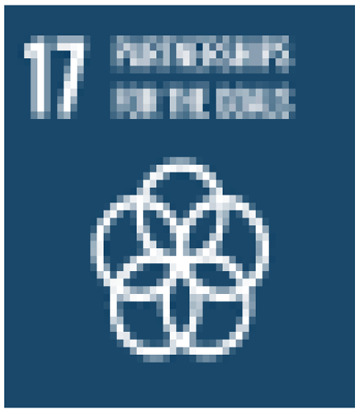	Partnerships for the goals	• Methodology enables knowledge sharing across international pharmaceutical regulatory bodies
		• Simple, cost-effective approach facilitates adoption in resource-limited settings
		• Promotes standardization and reproducibility in global pharmaceutical quality control

The triangular pyramid that represents the NQS Index illustrates the vital balance between quality, sustainability, and necessity that is necessary for analytical chemistry excellence. The suggested method's excellent performance in every dimension is reflected in its placement close to the pyramid's summit.

#### 3.6.6 Comparative analysis of sustainability assessment tools

A robust sustainability evaluation in analytical chemistry necessitates a multifaceted approach, as no single tool can holistically capture all dimensions—environmental, operational, economic, and innovative. Accordingly, this study adopts an integrated multi-tool strategy, wherein each tool contributes unique perspectives and compensates for the limitations of others.

The NEMI provides rapid, qualitative screening through a four-quadrant pictogram system addressing persistence, bioaccumulation, toxicity (PBT), corrosiveness, hazard potential, and waste generation. While NEMI ensures regulatory conformity and identifies key environmental hazards, its binary scoring system lacks the granularity required for nuanced sustainability assessments.

GEMAM addresses NEMI's limited resolution by providing semi-quantitative evaluation through 21 parameters across six domains (sample, reagent, method, waste, instrumentation, and operator). Unlike earlier tools, GEMAM balances qualitative visualization with numerical scoring, offering a more actionable framework. However, it focuses exclusively on environmental sustainability and does not fully capture practical or economic dimensions.

CFA was incorporated to provide quantitative assessment by estimating greenhouse gas emissions in CO_2_ equivalents. It adds depth by considering energy usage, reagent transport, and waste management. Nevertheless, while CFA enhances environmental resolution, it excludes analytical performance, innovation, or usability metrics.

To bridge environmental and practical domains, BAGI evaluates ten real-world implementation factors—including sample throughput, reagent consumption, automation, and cost—yielding a geometric mean score reflecting operational efficiency. Though BAGI effectively assesses practical applicability, it does not measure environmental burden or analytical innovation, necessitating complementary tools.

Recognizing the importance of methodological novelty, VIGI was applied to uniquely quantify the innovative character of analytical methods across ten dimensions, including data processing, automation, interdisciplinary integration, and white analytical chemistry principles. It captures the often-overlooked influence of innovation on sustainability but does not account for environmental safety or practical feasibility.

To unify environmental (green), practical (blue), and analytical performance (red) dimensions, the RGBfast model—a simplified but comprehensive adaptation of the original RGB framework—was employed. RGBfast evaluates six criteria spanning trueness, precision, detection limit, reagent hazard, throughput, and energy consumption. This model introduces the “whiteness” score, enabling simultaneous consideration of method quality and sustainability, serving as a convergence point for individual assessments.

Finally, the NQS Index synthesizes these dimensions within the framework of the UN Sustainable Development Goals (UN-SDGs). This final tier not only contextualizes the analytical method's performance and greenness but also aligns it with broader societal needs and environmental imperatives, including SDG 3 (Health), SDG 7 (Energy), and SDG 13 (Climate Action).

In summary, each tool fulfills a distinct role: NEMI, GEMAM, and CFA prioritize environmental aspects, BAGI addresses practical implementation, VIGI captures methodological innovation, RGBfast integrates performance with greenness and applicability, and NQS contextualizes all within global sustainability objectives.

Among these assessment tools, RGBfast and NQS emerged as the most integrative and diagnostically powerful frameworks. Nevertheless, no single tool is sufficient in isolation. Thus, a multi-tool composite strategy is indispensable for delivering a holistic sustainability profile. This integrated approach not only ensures balance across all sustainability dimensions but also supports informed, evidence-based decisions during method development and selection.

The consistent superiority of the proposed method across all assessment frameworks demonstrates how advanced spectrofluorimetric approaches can simultaneously address critical sustainability challenges while maintaining exceptional analytical performance. This approach establishes a new paradigm for sustainable pharmaceutical analysis that aligns scientific innovation with global sustainability imperatives through elimination of complex separation processes and minimization of resource consumption.

Future developments should aim to create unified platforms or hybrid metrics that simultaneously quantify environmental, analytical, economic, and societal dimensions, thereby advancing the field toward truly sustainable analytical science.

Overall, the results of this study demonstrate that PNK possesses highly favorable intrinsic fluorescence that can be markedly enhanced and stabilized in cetrimide micellar media, enabling sensitive and selective spectrofluorimetric quantification. Systematic optimization of solvent, pH, micellar conditions, and experimental parameters yielded a robust method characterized by nanogram-level detection limits, wide linear range, and excellent precision and recovery in both pharmaceutical formulations and spiked plasma. Mechanistic investigations clarified the underlying photophysical processes, confirming micellar incorporation, protection from quenchers, and lifetime extension as the basis of the observed signal amplification. Furthermore, the comprehensive sustainability evaluation highlighted the method's environmental safety, low carbon footprint, cost-effectiveness, practical feasibility, and innovation, establishing a favorable balance between analytical performance and green chemistry principles. Collectively, these findings confirm that the proposed micellar spectrofluorimetric method not only addresses analytical challenges associated with PNK determination but also contributes to advancing sustainable, high-throughput bioanalytical strategies, thereby supporting broader applications in pharmaceutical quality control, therapeutic monitoring, and clinical research.

## 4 Conclusion

This investigation presents a novel comprehensive exploration of PNK's intrinsic fluorescence properties for pharmaceutical analysis, establishing a novel, eco-friendly, and highly sensitive spectrofluorimetric methodology. The developed approach represents a paradigm shift in pharmaceutical analysis by eliminating the need for chemical derivatization or fluorescent labeling while achieving exceptional analytical performance through cetrimide-enhanced micellar systems.

The optimized method demonstrates remarkable analytical merit with a linear dynamic range of 100–800 ng mL^−1^ (*r*^2^ = 0.9999), exceptional sensitivity, and optimal fluorescence response at excitation/emission wavelengths of 286/418 nm. The 2% w/v cetrimide in ethanol micellar medium proved instrumental in significantly enhancing fluorescence intensity while maintaining method simplicity and environmental compatibility.

Complete validation following ICH Q2(R1) guidelines confirmed the method's robustness across all critical performance parameters. The methodology demonstrated excellent accuracy (recovery rates: 98.5–101.2%), precision (RSD ≤ 2.0%), and specificity, with successful application to pharmaceutical formulations (Onon® capsules), biological matrices (human plasma), and content uniformity assessments according to USP standards. The method's versatility and reliability across diverse sample types underscore its practical usefulness in pharmaceutical quality control and bioanalytical applications.

The sustainability assessment represents a groundbreaking contribution to green analytical chemistry, employing an integrated multi-tool evaluation framework that comprehensively addresses environmental, economic, and innovative dimensions. The method achieved exceptional scores across all assessment tools: complete NEMI compliance (four green quadrants), outstanding GEMAM score (7.487/10), minimal carbon footprint (0.002 kg CO_2_ per sample), excellent BAGI score (90.00), high VIGI innovation index (80.00), superior RGBfast whiteness score (85.00), and remarkable NQS Index (89%). This comprehensive evaluation demonstrates 80–93% reduction in carbon footprint compared to conventional chromatographic methods, with significant contributions to 11 UN Sustainable Development Goals.

The economic advantages are equally compelling, with per-sample analysis costs of $0.95 compared to $5–15 for conventional chromatographic approaches, representing substantial cost savings without compromising analytical quality. The method's rapid analysis time (<5 minutes), minimal sample preparation requirements, and compatibility with existing laboratory infrastructure further enhance its practical appeal for routine pharmaceutical analysis.

The innovative integration of intrinsic fluorescence detection with micellar enhancement addresses critical limitations of existing analytical approaches while developing white analytical chemistry's fundamentals. By limiting waste production, cutting energy use, and getting rid of dangerous organic solvents, this methodology aligns with contemporary sustainability imperatives while maintaining superior analytical performance.

The successful implementation across pharmaceutical, biological, and content uniformity applications validates the method's broad applicability and reliability. The comprehensive sustainability profile establishes this approach as a benchmark for environmentally responsible pharmaceutical analysis, demonstrating how innovative analytical strategies can simultaneously enhance performance while reducing environmental impact.

This work contributes significantly to the advancement of sustainable pharmaceutical analysis by providing a robust, cost-effective, and environmentally benign alternative to conventional separation-based methods. The methodology's exceptional performance across analytical, environmental, and economic dimensions makes it a useful instrument for pharmaceutical quality control laboratories seeking to adopt more sustainable analytical practices.

Nonetheless, some limitations should be noted. The method requires micellar optimization when extended to structurally different drugs or highly complex biological matrices, as analyte–micelle interactions are compound dependent. Furthermore, large-scale validation across diverse patient populations and clinical trial samples will be needed to fully establish robustness under real-world conditions.

Future research directions should focus on extending this intrinsic fluorescence approach to other pharmaceutical compounds with similar fluorescent properties, developing automated systems for enhanced throughput, and investigating potential applications in emerging pharmaceutical formulations and complex biological matrices. The established framework provides a foundation for continued innovation in sustainable pharmaceutical analysis, contributing to the overarching objective of ecologically conscious analytical science.

The demonstrated success of this spectrofluorimetric methodology validates the potential of intrinsic fluorescence-based approaches in pharmaceutical analysis and establishes a new standard for sustainable, high-performance analytical methods in the pharmaceutical industry.

## Ethics approval and consent to participate

This study did not involve direct human participation or the collection of new human samples. Drug-free human plasma was commercially obtained from the Egyptian Holding Company for Biological Products and Vaccines (VACSERA), Giza, Egypt. According to article 3 of the Egyptian Clinical Research Law no. 214 of 2020 and VACSERA's institutional biosample usage policy,^36–38^ the use of anonymized, commercially available plasma for non-interventional, analytical method development does not require prior ethical approval or informed consent. All procedures were conducted in accordance with the ethical standards of the declaration of Helsinki.

## Author contributions

O. A. supervision, methodology, writing of the original draft and investigation. L. A. A. writing of the original draft, interpretation of data and figures preparation. M. K. H. supervised analysis procedures and carried out sample preparation. G. M. writing the original draft and visualization. A. E. F. A. methodology, design of the work, investigation, writing of the original draft, and writing review and editing. All the authors read, reviewed, and approved the manuscript.

## Conflicts of interest

The authors state that they are not aware of any financial or interpersonal issues that might have affected the study that was the subject of this work.

## Supplementary Material

RA-015-D5RA05505A-s001

## Data Availability

The corresponding author will provide the datasets created and/or analyzed during the current study upon reasonable request. Supplementary information: the graphical abstract, highlights, and Tables S1–S3. See DOI: https://doi.org/10.1039/d5ra05505a.
