# Supplementary material for: First-time exploitation of Pranlukast's intrinsic fluorescence: a novel cetrimide-enhanced spectrofluorimetric platform for pharmaceutical, plasma, and content uniformity analysis
Source: RSC Adv. 2025 Oct 9;15(45):37684–704. doi: 10.1039/d5ra05505a (PMC12510162; doi:10.1039/d5ra05505a)
Supplement: RA-015-D5RA05505A-s001 [file RA-015-D5RA05505A-s001.pdf]

This journal is © The Royal Society of Chemistry 2025

Supplementary Tables

Table S1: Robustness of the proposed spectrofluorimetric method for PNK determination.

Parameter Varied	Nominal Condition	Modified Condition	Recovery (%) ± SD	%RSD
Excitation wavelength	286 nm	284 nm	99.32 ± 0.68	0.69
		288 nm	100.15 ± 0.74	0.74
Cetrimide concentration	2.0% w/v	1.9% w/v	99.67 ± 0.81	0.81
		2.1% w/v	100.23 ± 0.65	0.65
Ethanol proportion	<2% v/v	1.8% v/v	99.54 ± 0.72	0.72
		2.2% v/v	100.41 ± 0.77	0.77

Table S2: Stability of PNK in plasma under different conditions.

Stability Condition	Storage/Handling	Recovery (%) ± SD	%RSD
Short-term stability	6 h at room temperature	98.76 ± 0.84	0.85
Long-term stability	4 weeks at –80 °C	101.12 ± 0.91	0.90
Freeze–thaw stability	3 cycles (–80 °C to RT)	97.95 ± 1.03	1.05

Table S3: Side-by-side comparison of PNK analytical methods

Criteria	Kalyankar et al. 2021 (Stability-indicating RP-HPLC)	Marchese et al. 1998 (LC–MS/MS)	Lohar et al. 2019 (RP-HPLC–PDA)	Gajanan et al. 2018 (UV–Vis)	Present work 2025 (Micellar spectrofluorimetry)
**Matrix validated**	Laboratory mixture only	Human plasma	Rat plasma	Formulation only	Capsules **and** spiked plasma
**Technique**	RP-HPLC (stability-indicating)	LC–MS/MS + on-line SPE	RP-HPLC–PDA (LLE)	UV–Vis spectrophotometry	Micellar-enhanced native fluorescence
**Linear range**	100–500 ng mL^{-1}	10–2000 ng mL^{-1}	100–1600 ng mL^{-1}	10–50 µg mL^{-1}	**100–800 ng mL^{-1}**
**LOD / LOQ**	6.28 / 19.03 ng mL^{-1}	10 / 1 ng mL^{-1} (metabolites)	100 ng mL^{-1} (LLOQ)	490 / 1510 ng mL^{-1}	**9.87 / 29.91 ng mL^{-1}**
**Precision (RSD%)**	Intra: 0.13–0.55Inter: 0.21–0.74	< 12	2.3–9.9	≤ 1.3	**0.44–0.74**
**Recovery / Accuracy (%)**	99.41–99.72	Acceptable QC	90–91	98–100	**Capsules: 99.43–100.49Plasma: 97.87–99.29**
**Environmental profile**	85 % acetonitrile, no green metrics	High solvent waste, no green metrics	Organic extraction solvents, no green metrics	Requires ethanol, no assessment	**Fully green (NEMI all-green, GEMAM 7.487, RGBfast 85, CFA 0.002 kg CO_2/sample)**
**Instrumentation cost**	Moderate–high (HPLC)	**Very high (LC–MS/MS)**	Moderate (HPLC)	Low (UV)	**Low (standard fluorimeter)**
**Throughput / Speed**	10 min runs	Multi-step SPE + MS run	~30 min runs	Fast but low sensitivity	**Rapid, near-instant readings**
**Selectivity / Stability-indicating**	Stability-indicating for mixtures	Excellent selectivity for metabolites	Not stability-indicating	Poor selectivity, excipient interference	**Sufficient selectivity with micellar enhancement; can complement quick forced-degradation screens**
**Overall limitations**	Costly instrumentation, toxic solvent, not plasma-validated, slower throughput	Extremely expensive, complex prep, high waste, inaccessible for routine QC	Moderate sensitivity, long runs, extraction solvents, no stability indication	Extremely poor sensitivity, narrow range, unsuitable for plasma, no degradation separation	**Minor: requires micellar optimization for unusual matrices**
**Overall strengths**	Robust, precise, stability-indicating	Ultra-sensitive, good for PK	Acceptable for preclinical bioanalysis	Very simple, inexpensive	**Comparable sensitivity to LC–MS/MS, plasma validation, fastest, lowest cost, environmentally superior, first native fluorescence method for pranlukast**
